# Mavacamten inhibits myosin activity by stabilizing the myosin interacting-heads motif and stalling motor force generation

**DOI:** 10.1126/sciadv.aea9335

**Published:** 2026-04-29

**Authors:** Sean N. McMillan, Jaime R. T. Pitts, Bipasha Barua, Donald A. Winkelmann, Charlotte A. Scarff

**Affiliations:** ^1^ Discovery and Translational Science Department, Leeds Institute of Cardiovascular and Metabolic Medicine, School of Medicine, Faculty of Medicine and Health, University of Leeds (UoL), UK.; ^2^ Astbury Centre for Structural Molecular Biology, UoL, UK.; ^3^ School of Molecular and Cellular Biology, Faculty of Biological Sciences, UoL, UK.; ^4^ Department of Pathology and Laboratory Medicine, Robert Wood Johnson Medical School, Rutgers University, Piscataway, NJ 08854, USA.

## Abstract

Most sudden cardiac deaths in young people arise from hypertrophic cardiomyopathy, a genetic heart muscle disease. Treatment has until recently been limited to symptomatic relief or invasive procedures. Small-molecule modulators of cardiac myosin are promising therapeutic options to target disease progression. Mavacamten, the first Food and Drug Administration–approved example, has an unclear mechanism. To address this, we solved cryo–electron microscopy (cryo-EM) structures of beta-cardiac heavy meromyosin in three adenosine 5′-diphosphate and inorganic phosphate (ADP.P_i_)–bound states, the primed motor domain with and without mavacamten and the autoinhibited interacting-heads motif (IHM) with mavacamten, to 2.9, 3.4, and 3.7 Å global resolution, respectively. Together with quantitative cross-linking mass spectrometry analysis, these structures reveal how mavacamten inhibits myosin. Mavacamten stabilizes ADP.P_i_ binding, stalling the motor domain in a primed state, reducing motor dynamics required for actin-binding cleft closure, and slowing progression through the force generation cycle. These effects propagate within the two-headed molecule, stabilizing the IHM through increased motor-motor contacts. While this promotes diastolic relaxation, it also reduces systolic contractile output.

## Teaser

Mechanism of the myosin inhibitor mavacamten is revealed by cryo-EM and cross-linking studies.

## INTRODUCTION

Hypertrophic cardiomyopathy (HCM) (see table S1 for full list of abbreviations) affects at least 1 in 500 people and is the most common cause of heart failure in the young (*1*). It is a genetic disease of the sarcomere, with ~40% of known disease-causing mutations found in the beta-cardiac myosin (βCM) heavy chain gene (*MYH7*) (*2*) and causing ~30% of disease cases (*3*). The exact disease mechanism(s) for HCM remains unclear. Treatment typically involves symptomatic relief, with beta or calcium channel blockers, or invasive procedures, such as septal myectomy, which do not address the root cause of disease (*4*). More recently, small-molecule treatments have been developed to directly modulate βCM force production (*5*) and tackle disease progression. Mavacamten (*6*, *7*) is the first of these to be Food and Drug Administration approved, with many others in clinical trials (*8*, *9*). However, our understanding of the molecular mechanism of mavacamten is limited, restricting our understanding of its functional effects within disease.

βCM is the molecular motor responsible for force generation in cardiac ventricular tissue (*10*), fueled by adenosine 5′-triphosphate (ATP) and through its interaction with filamentous actin. A single βCM molecule is composed of two heavy chains and four light chains, of which two are essential (ELC) and two are regulatory (RLC). Each heavy chain consists of an N-terminal globular motor domain, light chain binding domain (LCD) (where one ELC and one RLC bind) followed by an alpha helical region through which two heavy chains dimerize to form a coiled coil. The coiled coil is further divided into subfragment 2 (S2) and the filament-forming light meromyosin (LMM). Each motor domain is divided into four subdomains, the N-terminal, lower 50-kDa (L50), upper 50-kDa (U50), and the converter that together with the LCD forms the lever (*11*, *12*).

Myosin molecules form bipolar filaments, with their LMM tails in the filament backbone and their paired motor domains on the filament surface (*13*, *14*). To drive contraction, they work in concert, interacting with actin to generate force transmitted by their levers in response to nucleotide and actin-binding state (*12*). The motor domain together with the lever, collectively termed S1 or a myosin head, as well as cardiac heavy meromyosin (cHMM) (myosin lacking its LMM region), are competent to produce force and thus frequently studied to understand the myosin mechanochemical cycle.

Upon ATP binding, a myosin head undergoes lever priming and ATP hydrolysis, to form a primed conformation (*15*, *16*). In this adenosine 5′-diphosphate and inorganic phosphate (ADP.P_i_)–bound state, myosin has an open actin-binding cleft between the U50 and L50 domains and can only interact with actin weakly. Conformational change within the motor leads to stronger actin-binding, cleft closure, P_i_ release, and myosin lever swing (powerstroke), generating force (*17*, *18*). ADP release from the motor results in a further, more minor shift in lever position (*19*). Rebinding of ATP opens the actin-binding cleft, disassociating the motor from actin, and the cycle starts again (*15*). Phosphate release is the rate limiting step for βCM in the absence (basal turnover) of actin, while in the presence of actin phosophate release is fast and coupled to force generation (*20*, *21*).

Within the heart, muscle contraction and thus force production are tightly regulated by two distinct mechanisms (*22*). Actin thin filament regulation acts as an on-off switch, controlling when contraction can occur through intracellular calcium levels, dictating when sites on actin are available for myosin binding (*23*). Myosin thick filament regulation fine-tunes the force output of individual thick filaments by controlling the number of motors available to produce force within the filament (*24*). To do this, βCM can form a sequestered state outside of the force generation cycle called the interacting-heads motif (IHM).

The IHM forms through the asymmetric interaction of two ADP.P_i_-bound primed motors from the same molecule folding down against their S2 coiled coil (*13*, *14*). Within the IHM, one of the heads [termed the blocked head (BH)] is blocked from interacting with actin as its actin-binding cleft is sequestered by the converter of the other head [termed the free head (FH)]. In filaments, the IHM state is further stabilized through interactions with the thick filament backbone, titin, and myosin-binding protein C (*13*, *14*).

In HCM, it has been hypothesized that some mutations elicit their effect by increasing the number of βCM molecules available to interact with actin and produce force (*25*, *26*), while others may directly affect force generation (*27*, *28*). In both cases, this can result in diminished relaxation and the diastolic dysfunction observed clinically (*29*). Thus, HCM may potentially be treated by therapeutics, which either inhibit myosin activity or increase formation of an off-state.

Mavacamten is a cardiac myosin allosteric inhibitor of sarcomeric force production, reducing cardiac contraction in animal models, isolated cells, and muscle fibers (*30*). It acts on basal and actin-activated adenosine triphosphatase (ATPase) activity by inhibiting P_i_ release and stabilizes an autoinhibited off-state (*31*), reducing the number of motors functionally available to generate contractile force (*30*). Although mavacamten can stabilize an autoinhibited off-state (*31*), the detailed structural nature of this state, the mechanism of this stabilization, and how it inhibits P_i_ release are unclear (*32*).

Several recent studies have begun to build on biochemical observations and reveal the structural mechanism of mavacamten. Three cryo–electron microscopy (cryo-EM) structures have resolved the human cardiac thick filament, two in the presence (*13*, *14*) and one in the absence of mavacamten (*33*). These structures demonstrated that mavacamten stabilizes the IHM but do not have sufficient resolution (ranging from ~20 to 6 Å) to observe the underlying mechanism. In addition, a recent crystal structure of a bovine S1 fragment complexed with ADP and beryllium fluoride (ADP.BeFx) and mavacamten revealed the mavacamten binding site and that it restrains the lever (*34*). The authors used molecular dynamic simulations to investigate how mavacamten inhibits myosin activity and proposed that mavacamten binding alters the L50 domain actin-binding interface to form a motor incompetent for force generation (*34*). However, BeFx is a nonhydrolyzable ATP analog (*35*), which blocks myosin force generation, such that this proposed mechanism cannot and does not explain how mavacamten inhibits Pi release.

Here, we performed a comprehensive analysis of the effects of mavacamten on cardiac myosin, from in vitro motility assays to structure determination by cryo-EM and cross-linking studies using mass spectrometry. We show how mavacamten allosterically stabilizes the IHM and inhibits Pi release from individual motor domains. We see no evidence for a substantially altered L50 domain structure or destabilization of the L50 domain helix-loop-helix actin-binding interface upon mavacamten binding, as recently proposed (*34*). In the context of the thick filament structures (*13*, *14*, *33*), as well as recent observations made on the actin activation of myosin (*18*), our data allow us to present a full structural mechanism for mavacamten inhibition of myosin.

## RESULTS

### Mavacamten stabilizes an off-pathway stalled ADP.Pi state

We ensured that our cHMM construct was functionally active and inhibited by mavacamten, as expected, by testing its ability to drive actin filament movement by use of an in vitro filament gliding assay (*36*, *37*) in comparison to a single-headed myosin construct (cS1) (fig. S1 and table S2). The mavacamten concentration required to reduce motility to 50% [median inhibitory concentration (IC_50_)] was 0.14 μM for cHMM and approximately fourfold higher for cS1 (0.62 μM) [consistent with previous reports; (*30*)]. When filament movement was tracked over time in the presence of mavacamten, we observed that the number of moving filaments did not change for cS1 but decreased for cHMM in a dose-dependent manner (fig. S1D and movie S1), consistent with the cHMM construct forming the IHM and reducing the number of motors available to interact with actin and produce movement.

To corroborate this, we examined the ratio of open head to IHM cHMM molecules in the presence and absence of mavacamten (fig. S2) using a negative stain EM assay. We found that mavacamten significantly increased the percentage of cHMM molecules in the IHM when compared to cHMM alone (*P* = 0.0002, determined by an unpaired two-tailed Student’s *t* test with respect to the βCM control).

To understand how mavacamten elicits these effects, we used cryo-EM to solve the structure of the cHMM motor domain in an open heads conformation, in the presence (MD_mava_) and absence of mavacamten (MD), and the cHMM IHM conformation in the presence of mavacamten (IHM_mava_) (see Methods and figs. S3 to S5).

We preincubated cHMM with ATP to enable formation of open and IHM ADP.P_i_ states prior to the addition of mavacamten [or dimethyl sulfoxide (DMSO) as control], followed by subsequent cross-linking with bis(sulfosuccinimidyl)suberate (BS3) to stabilize the myosin conformations formed (fig. S6). We then optimized cryo-EM grid and vitrification conditions for imaging cHMM molecules either in the open state or in the IHM, which were then selected for downstream processing accordingly (see Methods).

**Fig. 1.**
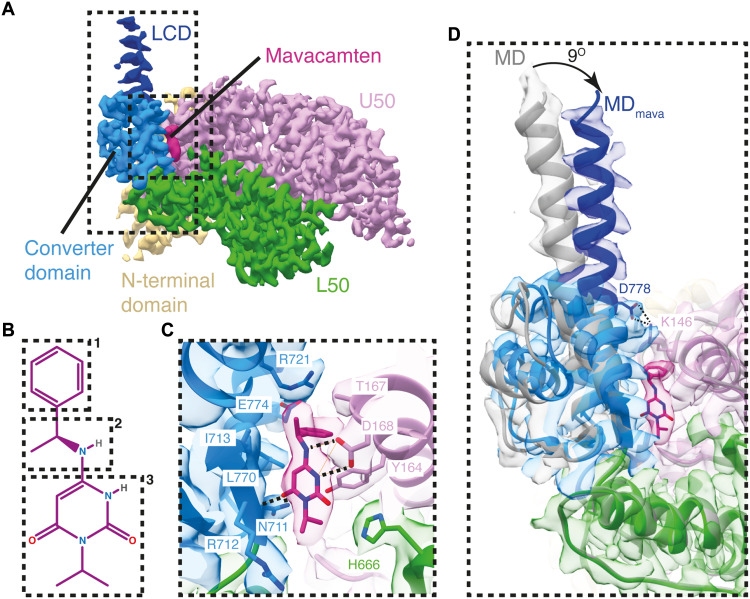
Mavacamten restrains the lever and D-helix. (**A**) Segmented cryo-EM map of the βCM motor in complex with mavacamten (MD_mava)_, split by subdomain (contour 0.6): N-terminal domain, beige; L50 subdomain, green; U50 subdomain, pink; converter domain, light blue; LCD, dark blue; and mavacamten, burgundy. (**B**) Phenyl (1), methylethyl ester (2), and isopropyl pyrimidinedione (3) moieties of mavacamten. (**C**) Mavacamten binding site highlighting key interactions: hydrophobic Arg^721^ (R721), Leu^770^ (L770), Ile^713^ (I713), His^666^ (H666), and Thr^267^ (T267); ionic Tyr^164^ (Y164); and hydrogen bonding from Asn^711^ (N711), Arg^712^ (R712), and Asp^168^ (D168). (**D**) Overlay of MD (gray) and MD_mava_ (colored and segmented) maps (contour MD: 0.5 and MD_mava_: 0.6) showing 9° shift in the lever due to mavacamten binding (global alignment) and formation of an ionic interaction Asp^778^-Lys^146^ (D778-K146).

The MD_mava_ and MD were resolved to 2.9 and 3.4 Å global resolution, respectively (fig. S4 and table S3). Both maps showed density for MgADP.P_i_ and a primed lever with associated ELC density, confirming they were in a primed conformation (fig. S7 and Fig. 1). The only discernible additional density within the MD_mava_ cryo-EM map in comparison to the MD map was observed between the converter and the U50 subdomain and could be attributed to the binding of a single mavacamten molecule (Fig. 1A and fig. S7, A and B). To interpret the cryo-EM density maps, we built pseudo-atomic models using a homology model and molecular dynamics–driven flexible fitting (table S4) (*38*).

Modeling enabled determination of the mavacamten binding pose. The mavacamten-protein interactions are predominantly hydrophobic, formed by the sidechain backbones of residues Arg^721^, Leu^770^ (L770), and Iso^713^ on the converter with isopropyl pyrimidinedione, methylethyl ester, and phenyl moieties of mavacamten (Fig. 1, B and C) as well as His^666^ on the L50 subdomain and Thr^167^ on the U50 subdomain with the isopropyl pyrimidinedione and phenyl moieties. These hydrophobic contacts are supported by an ionic interaction between Tyr^164^ (Y164) on the U50 subdomain and the isopropyl pyrimidinedione moiety and hydrogen bonding between Asn^711^ (N711), the backbone of Arg^712^ (R712) from the converter, and Asp^168^ from the U50 subdomain to the isopropyl pyrimidinedione and methylethyl ester moieties (Fig. 1, B and C). The same mavacamten binding pose and contacts were independently observed within the crystal structure of bovine S1 in complex with mavacamten [Protein Data Bank (PDB) ID: 8QYQ; figs. S8B and S9, A and B].

Binding of mavacamten rotates the lever 9° toward the U50 subdomain of the motor, perpendicular to the working stroke, when compared to the canonical primed MD conformation (Fig. 1D). This allows formation of a salt bridge between Asp^778^ (D778) and Lys^146^ (K146), creating additional communication between the lever and U50 subdomain (Fig. 1 and movie S2). Thus, mavacamten acts as a molecular glue, bonding the lever against the U50 subdomain.

**Fig. 2.**
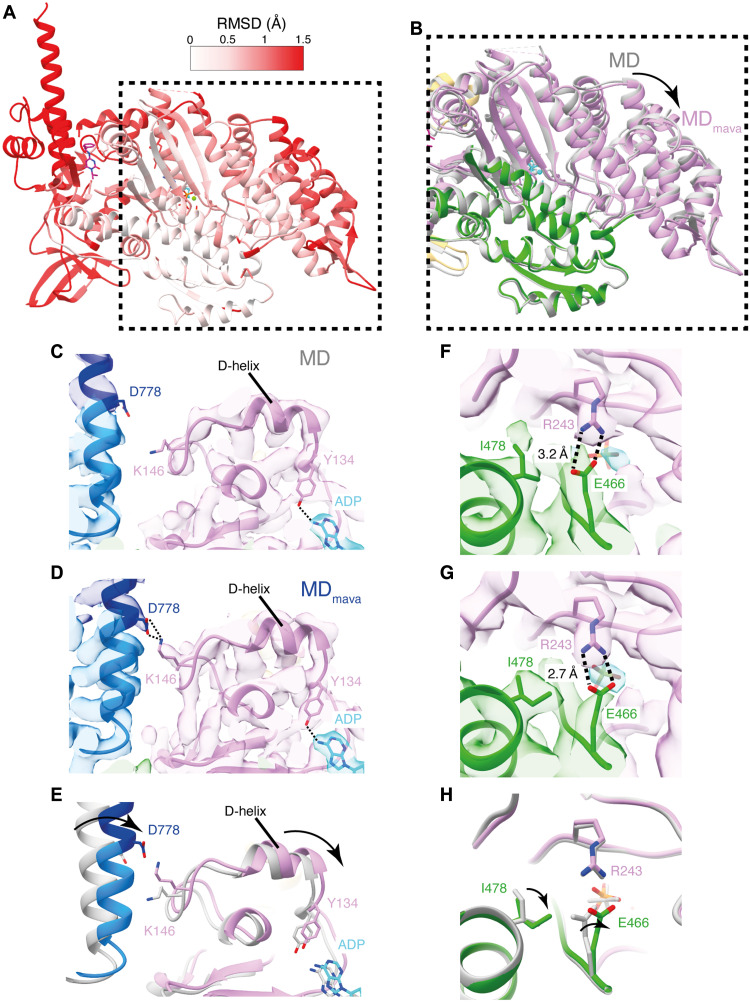
Mavacamten binding shifts the D-helix position stabilizing P_i_ in the active site. (**A**) Root mean square deviation (RMSD) comparison between MD and MD_mava_ structures colored on MD_mava_ model (aligned on the HLH). (**B**) Overlay of MD (gray) and MD_mava_ (colored), showing the subtle shift in the U50 (pink) toward the L50 (green). (**C** to **E**) Positioning of D778 and K146 in (C) MD, (D) MD_mava_, and (E) overlay MD (gray) and MD_mava_ (colored), highlighting the communication between lever and D-helix in MD_mava_. (**F** and **G**) MD and MD_mava_ structures in segmented maps respectively (contour MD: 0.6 and MD_mava_: 0.5), highlighting the change in back door Arg^243^-Glu^466^ (R243-E466) contact and Ile^478^ (I478) rotamer. (**H**) Overlay of MD (gray) and MD_mava_ (colored), highlighting the change in residue positioning.

To understand how mavacamten may affect the association of myosin with actin in the weakly bound ADP.P_i_ state, we aligned the MD and MD_mava_ structures on the L50 helix-loop-helix, the primary actomyosin-binding interface (*18*). Mavacamten binding subtly shifts the U50 subdomain relative to the L50 subdomain, pinching the actin-binding cleft in a motion distinct from cleft closure (Fig. 2, A and B, and movie S2). This may be driven by the increased communication between the lever and the U50 subdomain, which moves the D-helix toward the active site, altering the relative position of Tyr^134^ and nucleotide (Fig. 2, C to E), compressing the ADP.P_i_ binding site.

The shift of the U50 subdomain toward the L50 subdomain results in several rotamer changes around the “backdoor,” an ionic interaction between Glu^466^ (E466) and Arg^243^ (R243) that blocks the phosphate exit tunnel in the primed conformation. Ile^478^ adopts a rotamer conformation facing the backdoor, and the E466 side chain is rotated such that the distance between the R243 nitrogen (NH1) and E466 oxygen (OE1) is reduced from 3.2 to 2.7 Å (Fig. 2, F to H, and movie S2). This shows that in the presence of mavacamten, the back door interaction is stabilized, which may reduce the likelihood of P_i_ release.

These structural observations are not seen in the crystal structure of bovine S1 in complex with mavacamten (PDB ID: 8QYQ) (fig. S8) (*34*). This could potentially be due to crystal packing effects restricting protein conformation within the unit cell, use of bovine over human βCM, or the use of the nonhydrolyzable ATP analog ADP.BeFx in comparison to the native ADP.P_i_ ligand (fig. S9). Crucially, in the latter case, the beryllium blocks the catalytic site in a tetrahedral rather than penta-coordinated structure, stopping the motor from progressing through the catalytic cycle (*35*). Thus, our cryo-EM structures represent βCM motor domains with native ADP.P_i_ substrate and demonstrate how mavacamten stabilizes a stalled ADP.Pi state, providing structural evidence for the mechanism of inhibited P_i_ release (*31*, *34*, *39*).

The mavacamten-induced structural changes are directionally opposed to those observed upon actin activation (*18*), where the release of the P_i_ is accelerated ~100-fold. Recent structural observations for myosin-5 have shown that upon initial binding of myosin to actin, the U50 subdomain, particularly the D-helix, is cocked back toward the converter. This cocking back motion expands the nucleotide pocket destabilizing the free P_i_ and promotes cleft closure (*18*). Thus, we wondered whether mavacamten binding through cocking of the U50 subdomain forward, stabilizing ADP.P_i_ binding, may also reduce motor domain dynamics to limit cleft closure. This would slow the transition from weak to strong binding of myosin to actin in the mechanochemical cycle, which is needed to sustain force and enable P_i_ release.

### Mavacamten binding reduces myosin lever and actin-binding cleft dynamics

To investigate the effect of mavacamten on primed motor lever and actin-binding cleft dynamics, we used a quantitative cross-linking mass spectrometry (qXL-MS) approach. We used the MS-cleavable, amine and hydroxyl reactive cross-linker disuccinimidyl dibutyric urea (DSBU) to cross-link cS1 ADP.P_i_ in the absence and presence of mavacamten and then identified and quantified the cross-links obtained using an label-free qXL-MS comparative approach (see Methods) (*40*–*42*). The intermotor interactions found in cHMM could potentially obscure changes to the motor-lever interface. So, we chose to cross-link cS1, such that any differences observed would reflect relative changes in individual motor domain dynamics and not IHM formation. We identified 85 unique interpeptide cross-links, 67 intrapeptide cross-links, and 61 monolinks (where the cross-linking reagent had only attached to one reactive site) that changed in normalized signal intensity by at least twofold between the two conditions (see data S1 and S2 and fig. S10), indicating that adding mavacamten significantly affected cS1 conformational dynamics.

**Fig. 3.**
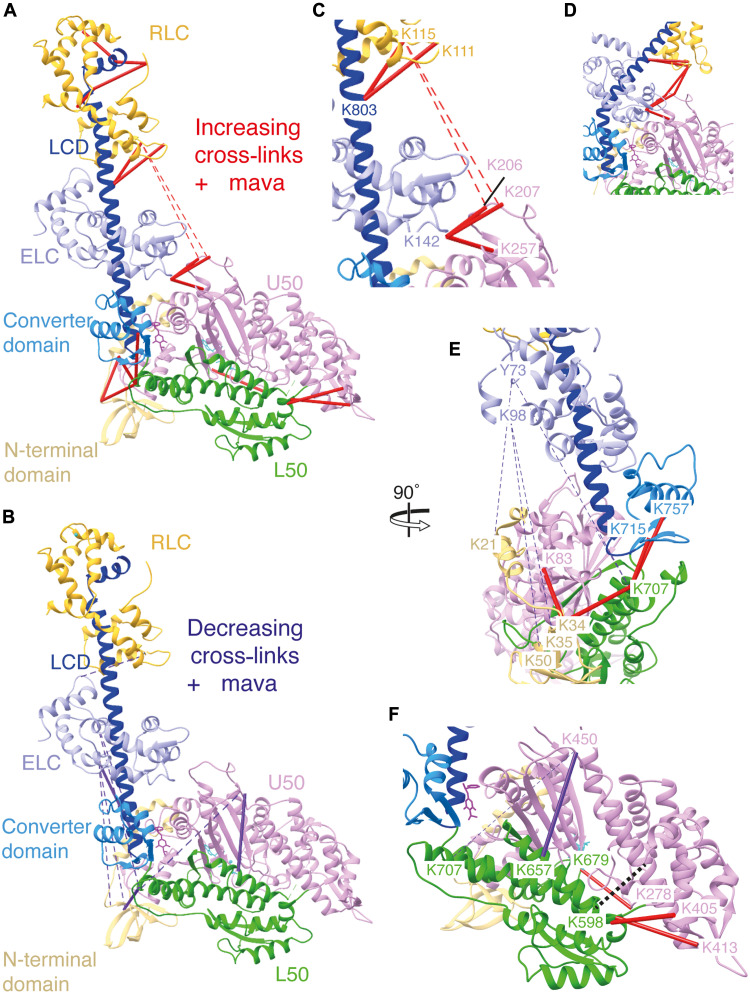
qXL-MS analysis of cross-links in the presence of mavacamten. Overview of significant cross-links (*P* < 0.05) observed to (**A**) increase (fold change >2) and (**B**) decrease (fold change <0.5) in the presence of mavacamten mapped onto the S1_mava_ model colored by subdomain: N-terminal domain, beige; L50, green; U50, pink; converter domain, light blue; LCD, dark blue; ELC, light purple; RLC, yellow; and mavacamten, burgundy. Solid lines denominate DSBU cross-links with a <30-Å Cα-Cα distance while dashed lines represent cross-links with a >30-Å Cα-Cα distance; red = increasing and purple = decreasing. (**C** and **D**) Magnified view of increasing cross-links between the light chains and U50 subdomain, with an (C) open head and (D) BH lever position. (**E**) Overview of cross-links showing increased stability of lever position in the presence of mavacamten. (**F**) Overview of cross-links showing changes in the U50-L50 position in the presence of mavacamten. Loop2 is represented by a dotted black line. Cross-links shown here are further described in table S5.

**Fig. 4.**
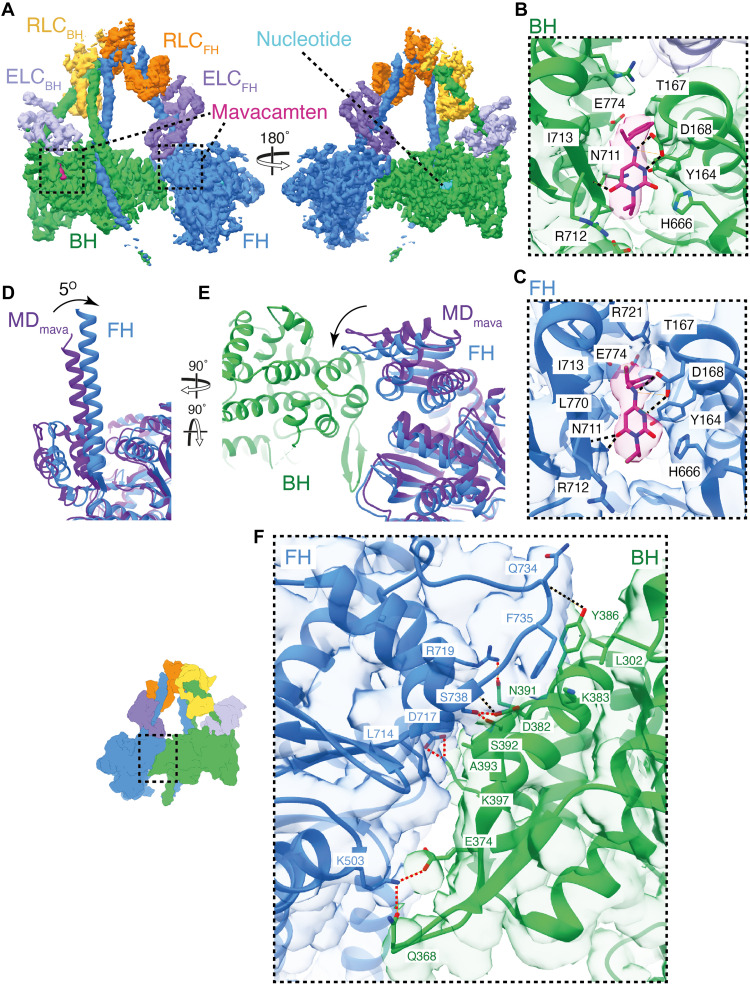
Mavacamten strengthens the motor-motor interface in the IHM by altering the FH lever conformation. (**A**) Segmented cryo-EM map of the IHM_mava_ structure colored by chain (contour: 0.08): BH, green; FH, blue; ELC_BH_, light purple; ELC_FH_, purple; RLC_FH_, orange; RLC_BH_, yellow; mavacamten, burgundy; and nucleotide, light blue. (**B** and **C**) The IHM_mava_ model fitted to the segmented map highlighting the BH and FH mavacamten pockets, respectively. (**D**) Side view of the FH lever (blue) overlaid on the MD_mava_ (dark purple), aligned on the motor Asp^3^-Pro^710^, highlighting the 5° shift of the lever. (**E**) Top-down view of (D) including the BH in green showing the movement of the FH converter. (**F**) Interaction interface between the U50_BH_-converter_FH_ (contour: 0.01), with H-bonds unique to the IHM_mava_ in red [compared to PDB ID: 8ACT; (*44*)].

To interpret the effects of mavacamten on human cS1 dynamics (Fig. 3), we generated human S1 models, S1_mava_ and S1 in the presence and absence of mavacamten, by superimposing the FH lever, and ELC and RLC from our IHM_mava_ model (see Fig. 4A) onto our MD_mava_ and MD structures, respectively, and replacing the mouse light chains found in the IHM for the human light chains primarily found in the cS1 construct (see Methods). We then annotated the models with interdomain cross-links that significantly increased or decreased in signal intensity twofold accordingly (*P* < 0.05 across three replicates) and measured their Cα-Cα distances (table S5). Cross-links within the accepted DSBU reaction distance of 30-Å Cα-Cα were expected (*43*). Comparatively, observation of cross-links with a Cα-Cα distance >30 Å indicated conformational dynamics that allowed the two reacting side chains to transiently come into range.

Thus, if a significant increase in a cross-link signal intensity in the presence of mavacamten was observed with the same Cα-Cα distance in both S1_mava_ and S1 models, then this indicated increased sampling of that conformational state and reduced dynamics, whereas an increase in signal intensity for a cross-link with a Cα-Cα distance >30 Å would suggest increased dynamics, as there was an increase in reactivity, e.g., increased time in which the two residues sampled a conformation outside our model in sufficient proximity to react.

To enable understanding of the changes in conformational dynamics that occurred upon the addition of mavacamten, we focused on interpreting cross-links that were interdomain, formed between individual motor subdomains, or the motor subdomains and human light chains. We identified 35 of these interdomain cross-links, of which we could annotate 27 on our models (Fig. 3, A and B, and table S5). The remainder could not be annotated as they were located within unmodeled or flexible regions, such as the N-terminal extension of the ELC or myosin loop2.

In the presence of mavacamten, the number of cross-links (<30-Å Cα-Cα distance) between the U50 domain and ELC increased significantly (Fig. 3, A and C, and table S5). This was accompanied with a decrease in the ELC intrapeptide cross-link Lys^142^-Thr^147^ (K142-T147). This is consistent with the lever being pulled in toward the U50 domain when mavacamten binds, as observed in our cryo-EM structures (Fig. 1). Cross-linking was also increased between the RLC and the LCD [Lys^111^/Lys^115^ (K115_RLC_) to Lys^803^ (K803_LCD_); Lys^91^/Lys^165^ (K165 _RLC_) to Lys^825^ (K825_LCD_); and Lys^62^ (K62)/K165_RLC_ to Lys^835^ (K835_LCD_)], and the RLC and loop1 [K115 to Lys^206^/Lys^207^ (K206/K207)], indicating increased stability of the lever. The RLC-loop1 cross-links had a Cα-Cα distance >30 Å in our S1 models but would have a Cα-Cα distance <30 Å if the lever was bent at the pliant point, forming the IHM BH lever position, consistent with the mavacamten-promoting formation of this state (Fig. 3D and table S5).

In the presence of mavacamten, cross-links between the base of the converter (CON) and the L50 domain [Lys^707^-Lys^715^ (K707_L50_-K715_CON_) and K707_L50_- Lys^757^ (K757_CON_); Fig. 3E] increased significantly, without an increase in Cα-Cα distances in our S1 models, indicating increased stability of L50-converter position. Exploratory cross-links (Cα-Cα distance >30 Å) between the ELC and N-terminal domain (NTD) [Tyr^73^-Lys^21^ (Y73_ELC_-K21_NTD_) and Lys^98^ (K98_ELC_) to Lys^21^/Lys^35^/Lys^50^ (K21/K35/K50_NTD_)], and ELC (Y73) and converter (K707), were significantly decreased in the presence of mavacamten (Fig. 3, B and E). This again suggests that the lever position is more stable with mavacamten bound, exploring a narrower range of primed conformations and preventing sampling of states that would allow these cross-links to form.

When considering actin-binding cleft dynamics, the cleft is ordinarily thought to open and close rapidly on the microsecond timescale (*16*) but with an equilibrium strongly favoring the open cleft position and retention of P_i_ in the active site. In the presence of mavacamten, cross-links (<30-Å Cα-Cα distance) between the HCM loop in the U50 domain (Lys^405^ and Lys^413^) and the strut (Lys^598^) within the L50 domain (Fig. 3F and table S5), bridging the actin-binding cleft, increased in signal intensity. There was also a significant increase in the Lys^278^_U50_ to Lys^679^_L50_ cross-link, on the opposite side of the actin-binding cleft. This suggests that cleft dynamics are reduced when mavacamten binds, enabling these cross-links to form more readily. This reduction in cleft dynamics may help prevent the weak-to-strong binding transition, inhibiting phosphate release and effective actomyosin cross-bridging.

The cross-links Lys^633^-Lys^657^ (K633_loop2_-K657_L50_) and Lys^450^ (K450_U50_)–K633_loop2_ (not modeled) also significantly increased, alongside a reciprocal decrease in the cross-link K450_U50_-K657_L50_ in the presence of mavacamten. Considered alongside the large increase of cross-links within loop2 (see data S1), observed upon the addition of mavacamten, this suggested that drug binding also stabilizes the conformation of loop2, enabling K657 to more readily cross-link with K633 than with K450.

The exploratory cross-link (>30-Å Cα-Cα distance) K450_U50_-K707_L50_ (table S5) was also decreased in signal intensity in the presence of mavacamten, again indicating reduced motor domain dynamics in the presence of the drug.

Notably, two cross-links within loop3 of the L50 had a large increase in signal intensity in the presence of mavacamten [Lys^565^(K565)–Lys^572^(K572) and Lys^570^(K570)–K572; data S1]. This is beautifully explained when we examine the hydrogen bonding network of loop3 within the MD and MD_mava_ structures, respectively (see fig. S11, A and B). K565 and K570 are both solvent exposed within both structures, but in the presence of mavacamten, an ionic interaction between Asp^469^ and K572 is lost, and instead, Asp^587^ interacts with Arg^567^ (see fig. S11C). This leaves K572 without a binding partner, making it available for cross-linking to K565 and K570 and accounting for the dramatic change in reactivity observed.

Although the rearrangement of this hydrogen bonding network may increase the dynamics of loop3 in the presence of mavacamten, there is a corresponding decrease to the cross-link Lys^559^(K559_L50_)–K565_L50_. This allows us to reasonably conclude that the localized increase in dynamics of loop3 is due to allosteric communication and not a wholesale increase of dynamics within the L50. Corroborating this, we see no evidence within the cryo-EM density maps for a decrease in stability of the helix-loop-helix motif (HLH) in the presence of mavacamten (fig. S4, L and M). This suggests that mavacamten does not have a direct effect on myosin weakly binding to actin, through disordering of the HLH, as proposed by Auguin *et al.* (*34*), but affects the weak-to-strong binding transition.

In addition to changes in cross-linking, we also observed changes to monolink signal intensity, where the cross-linker reacts with a residue and solvent. Monolinks for the ELC residues Tyr^129^ and K142 and LCD residue K803 decrease in the presence of mavacamten as the ELC and LCD residues are instead able to cross-link with the U50 and RLC, respectively (see data S1). The most significant decrease in monolinking in the presence of mavacamten was to Y164, not unexpected given that mavacamten directly interacts with this residue upon binding and so prohibits it from reacting with free cross-linker.

Together, our combined cryo-EM and qXL-MS structural analysis suggests that mavacamten stabilizes ADP.P_i_ binding and limits actin-binding cleft closure, allowing weak binding but inhibiting the weak-to-strong binding transition, slowing phosphate release and force generation. Thus, mavacamten stabilizes an off-pathway stalled myosin motor conformation that can weakly interact with actin but is unable to progress through the mechanochemical cycle.

### Mavacamten restrains the IHM through stabilization of the FH motor domain

To understand how mavacamten stabilizes the IHM, we used cryo-EM to solve the structure of IHM_mava_ to a global resolution of 3.7 Å (fig. S5 and Fig. 4A) and compared it to our MD_mava_ structure and to the mavacamten-free folded-back state [IHM; (*44*)] previously reported (PDB ID: 8ACT) (*44*). The overall appearance of the IHM_mava_ is consistent with the IHM structure. However, mavacamten binding induces distinct conformational changes within the motor domain of the BH and FH, respectively.

Mavacamten is bound to both the BH and FH of the IHM_mava_ (Fig. 4, B and C). The FH mavacamten binding site is compressed, with the lever domain lying even closer to the U50 than in the MD_mava_ structure (fig. S12), yet it maintains all interactions previously observed (Fig. 4C). Conversely, due to formation of the BH lever conformation (shown in Fig. 3D), the mavacamten BH binding site is expanded (fig. S12) compared to the MD_mava_ structure, and the interaction of mavacamten with N711 and L770 is lost (Fig. 4B). This suggests that mavacamten may have a greater effect on IHM_mava_ stabilization through its interaction with the FH.

The structural changes caused by mavacamten in the FH are the most prominent and provide a mechanism through which allosteric IHM stabilization occurs. Comparison of the MD_mava_ and IHM_mava_ FH, by alignment on the main body of the motor (residues 3 to 710), shows that the IHM_mava_ FH lever adopts a much sharper angle than in the MD_mava_ structure, rotating 5° perpendicular to the lever swing (Fig. 4D). If the same comparison is performed between the IHM (*44*) FH and IHM_mava_ FH, a rotation of 9° is observed in the same direction (fig. S13). The change in FH lever angle positions the converter closer to the BH, strengthening the U50_BH_-converter_FH_ interface.

As the FH converter now packs more tightly against the U50_BH_, several new interactions form, strengthening the existing interface between Asn^391^ (N391_BH_) and the backbone of Ser^738^ (S738_FH_) as well as Tyr^386^ (Y386_BH_) and Gln^734^ (Q734_FH_) (Fig. 4F) that were previously reported to occur in the IHM in the absence of mavacamten (*34*).

New interactions are formed between S738_FH_, Ser^392^_BH_ and Asp^382^ (D382_BH_), Asp^717^_FH_ and Lys^397^_BH_ as well as Arg^735^_FH_ and N391_BH_ in addition to a hydrophobic interaction between Ala^393^ (A393_BH_) and Leu^714^_FH_ (Fig. 4F and movie S3). The movement also results in rearrangement of Phe^735^_FH_, which now forms a hydrophobic interface with Lys383 (K383_BH_), Leu^302^_BH_, and Y386_BH_. (Fig. 4F and movie S3). Interactions are also altered at the HCM loop_BH_-transducer_FH_ interface with the formation of an ionic interaction between Asp^409^_BH_ and Arg^249^_FH_. Last, a new interaction interface between the U50_BH_ and the ELC_FH_ can form between Lys^611^_BH_ and Asp^143^_BH-ELC_ (movie S3).

In summary, the change in FH lever angle upon mavacamten binding provides increased structural rigidity to the IHM, through the increased contact between U50_BH_-converter_FH_ facilitating the generation of new interfaces, strengthening the motor-motor contact, and providing the structural mechanism for its allosteric stabilizing effects. This mechanism is supported by recent structural observations in which IHM crowns in a relaxed mavacamten-free thick filament structure were less ordered than in their mavacamten-bound counterparts, particularly in the FH of the disordered crown/crown 2 (*13*, *33*).

The BH conformation within the IHM_mava_ also deviates from that observed in both the MD_mava_ and the IHM (*44*) structures but only in the relative positioning of the U50 domain (see fig. S14). When the MD_mava_ is aligned on the L50_BH_ domain of the IHM_mava_, it is apparent that the U50_BH_ domain has moved across the L50_BH_ toward S2 (fig. S14, A and B). If the same alignment is performed with the IHM (*44*) and IHM_mava_, the same shift is observed (fig. S14, C and D). This conformational change in the U50_BH_ is needed to accommodate the altered converter_FH_ position upon mavacamten binding (fig. S14E). Thus, the conformational changes observed in the BH upon mavacamten binding are a consequence of its IHM stabilizing effect but not a contributor to IHM stabilization.

**Fig. 5.**
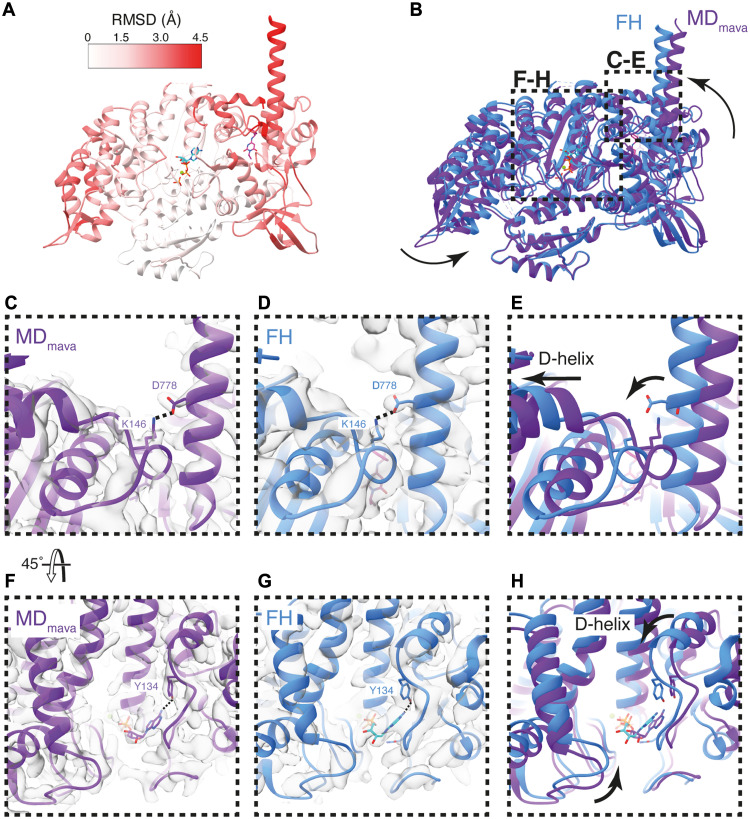
Mavacamten further inactivates the IHM FH. (**A**) RMSD comparison between MD_mava_ and IHM_mava_ FH aligned on the L50, colored on IHM_mava_ FH model. (**B**) Overlay of MD_mava_ and IHM_mava_ FH blue highlighting domain movements. (**C** to **E**) Structural comparison of lever and D-helix conformation showing D778-K146 coupling hydrogen bond. (C) MD_mava_ model and cryo-EM map (contour: 0.9), (D) IHM_mava_ FH model and cryo-EM map (contour: 0.15), and (E) model overlay colored as in (B). (**F** to **H**) Structural comparison of active site. (F) MD_mava_ model and cryo-EM map (contour: 0.9), (G) IHM_mava_ FH model and cryo-EM map (contour: 0.15), and (H) model overlay colored as in (B).

Along with stabilizing the motor-motor interface, mavacamten reduces the activity of the FH by restraining the nucleotide pocket. Comparison of the IHM_mava_ FH to our MD_mava_ structure and IHM (*44*) shows that the D-helix_FH_, is moved further toward the nucleotide pocket in the IHM_mava_ structure (Fig. 5, C to E, and fig. S13). The U50_FH_ has also shifted relative to the L50_FH_, further pinching the actin-binding cleft (Fig. 5, A and B, and fig. S13). The resulting shift of the D-helix_FH_ and surrounding loops further reduces the available space for ADP dynamics, increasing the stability of ADP.P_i_ within the active site (Fig. 5, F and G, and fig. S13). Once again, this is an opposite motion to that observed during actin activation (*18*).

**Fig. 6.**
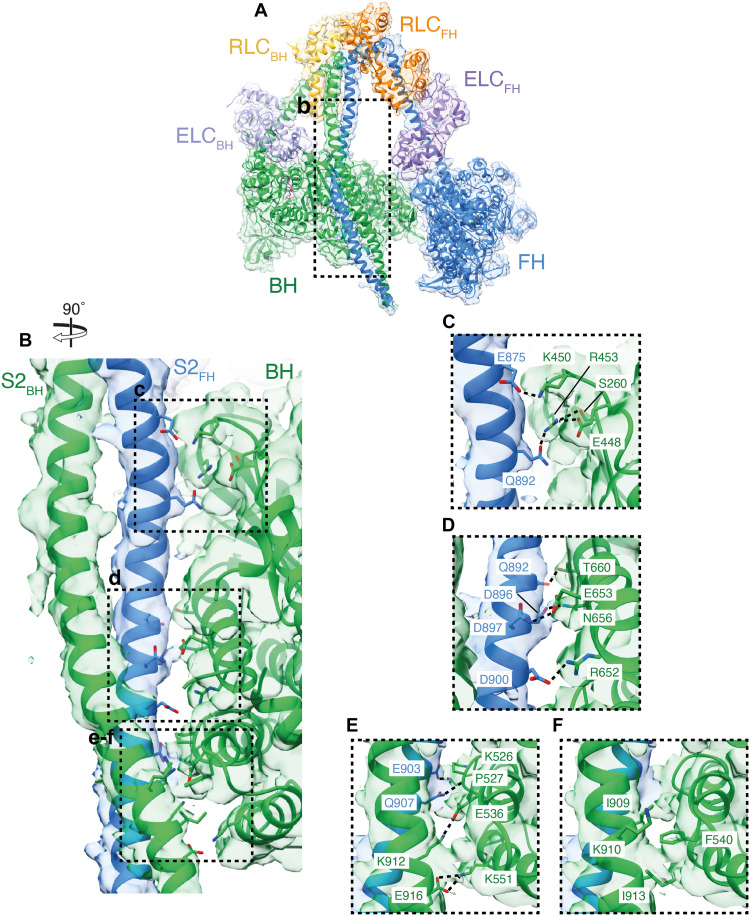
IHM_mava_ S2 interactions. (**A**) IHM_mava_ model in segmented cryo-EM map colored by chain (contour: 0.02): BH, green; FH, blue; ELC_BH_, light purple; ELC_FH_, purple; RLC_FH_, orange; RLC_BH_, yellow; mavacamten, burgundy; and nucleotide, light blue. (**B**) Overview of IHM_mava_ S2_BH_ interactions (modeled using molecular dynamics in ISOLDE) in segmented cryo-EM map (contour: 0.01): BH, green; FH, blue. (**C**) S2-HO linker hydrogen bonding: Glu^875^-Lys^450^ (E875_FH_-K450_BH_), Gln^892^-Arg^453^ (Q892_FH_-R453_BH_), and Arg^453^-Glu^448^ (R453_BH_-E448_BH_). (**D**) S2-W helix hydrogen bonding: Gln^892^-Thr^660^ (Q892_FH_-T660_BH_), Asp^896^-Asn^656^ (D896_FH_-N656_BH_), Asp^897^-Asn^653^ (D897_FH_-N653_BH_), and Asp^900^-Arg^652^ (D900_FH_-R652_BH_). (**E** and **F**) S2-HLH interactions (E) hydrogen bonding: Glu^903^-Pro^527^ (E903_FH_-P527_BH_) backbone, Gln^907^-Lys^526^ (Q907_FH_-K526_BH_), Lys^912^-Glu^536^ (K912_BH_-E536_BH_), and Glu^916^-Lys^551^ (E916_BH_-K551_BH_); (F) hydrophobic interactions: Ile^909^ (I909_BH_), Ile^913^ (I913_BH_), Phe^540^ (F540_BH_), and the aliphatic side chain backbone of Lys^910^ (K910_BH_).

Within the IHM_mava_ structure, the BH is further restrained by its interaction with S2 (Fig. 6). S2 predominantly interacts with the BH via the FH heavy chain directly strengthening the interaction between the two chains (Fig. 6A). The contact interfaces are predominantly charged interactions and can be split into three main regions on the BH motor: the OH loop, W-helix, and HLH (Fig. 6, B to F, and movie S3).

The S2 in our IHM_mava_ structure adopts a curved conformation more closely resembling that observed in the thick filament structures (*13*, *14*, *33*) over the mavacamten-free folded-back state structure (fig. S15) (*44*). This is unlikely to be an effect of mavacamten, but may be due to our use of a construct containing a longer native cHMM S2 domain sequence, extending well beyond the interface, rather than a short S2 sequence truncated at K942 and stabilized with a leucine zipper (*44*).

Examination of the S2-BH interface in our IHM_mava_ model suggests that it contains many more HCM mutation sites [Arg^453^→Cys, His, Ser, Leu (*45**–**48*), Arg^660^→Asn (*49*), Arg^652^→Gly (*50*), Lys^903^→Lys/Gly (*51*, *52*), Ile^909^→Met (*53*), and Ile^913^→Lys (*54*)] in addition to those previously reported in the IHM (*44*) [Lys^450^→Glu/Thr (*55*, *56*), Gln^882^→Glu (*57*), and Gln^892^→Lys (*58*)]. Thus, maintenance of this interface is likely crucial to regulate force production during cardiac contractions.

## DISCUSSION

**Fig. 7.**
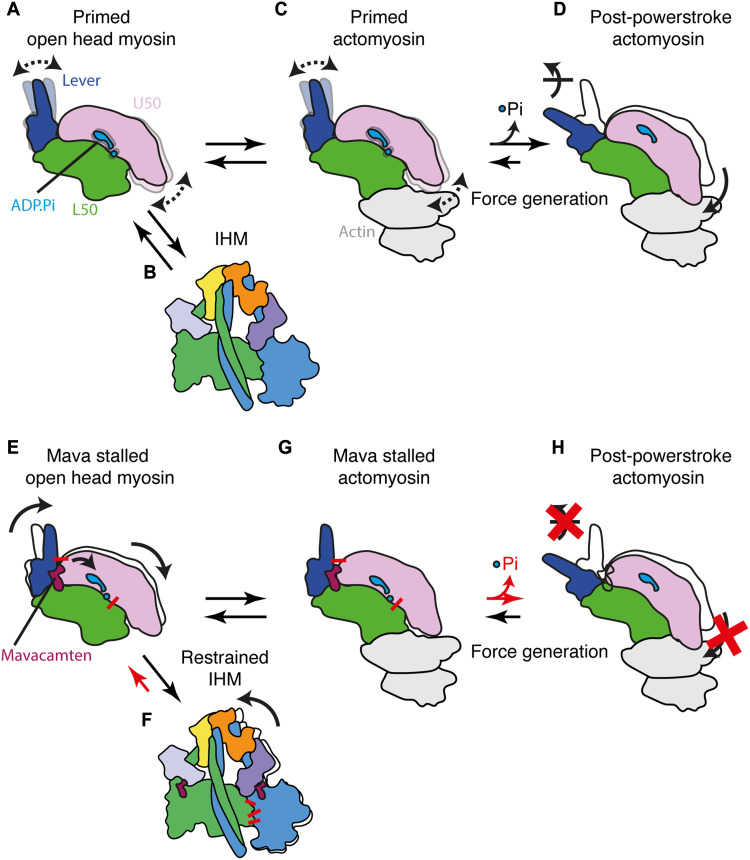
Overview of myosin force generation and mavacamten’s structural mechanism. (**A** to **D**) Schematic representation of βCM force generation: (A) Primed open head motor with both the lever and U50 dynamically exploring conformations able to transition to (B) the IHM preventing further force generation or (C) primed actomyosin. The primed actomyosin is initially weakly bound but rapidly proceeds through the weak-to-strong actin binding transition, releasing phosphate and undergoing the force generating powerstroke resulting in formation of (D) post-powerstroke actomyosin. (**E** to **H**) Schematic representation of βCM force generation in the presence of mavacamten: (E) Mavacamten stalled open head motor with restrained lever and shifted U50 stabilizing the P_i_, able to transition to (F) the restrained IHM and stabilized by increased motor-motor interactions. Mava stalled open head myosin may also interact with actin forming (G) stalled actomyosin. The stalled actomyosin does not readily undergo cleft closure (red X) preventing phosphate release and its transition through lever swing (red X) to (H) post-powerstroke actomyosin.

In this work, we have shown three high-resolution cryo-EM structures, two cHMM motor domains with and without mavacamten bound, alongside the cHMM IHM in complex with mavacamten, all containing the native substrate, ADP.P_i_. The open head cHMM motor domain structures in combination with our qXL-MS analysis and new insights into actin activation (*18*) allow us to present a mechanism through which mavacamten elicits its effect on the myosin mechanochemical cycle (Fig. 7).

Primed motors show lever and cleft dynamics that allow them to undergo basal ATPase activity (Fig. 7A) and weakly associate with actin. This can be regulated by sequestration of myosin molecules into the IHM, which substantially reduces ATPase activity (Fig. 7B). Upon weak binding of myosin to actin (Fig. 7C), cleft closure, P_i_ release, and powerstroke are accelerated, resulting in post-powerstroke actomyosin (Fig. 7D). Mavacamten stalls motor force generation by restraining lever position and inducing structural changes that stabilize ADP.P_i_ binding and limit cleft dynamics (Fig. 7E). This in turn increases the abundance of the IHM by strengthening interactions at the motor-motor interface (Fig. 7F). We find no evidence to support the proposal that mavacamten destabilizes the L50 to prohibit myosin weakly binding to actin (*34*). Instead, in disordered relaxed motors that can weakly associate with actin (Fig. 7G), we propose that mavacamten stabilizes ADP.P_i_ binding and reduces actin-binding cleft dynamics to limit cleft closure. This allows weak binding but inhibits the weak-to-strong actin binding transition that is required for progression through the mechanochemical cycle (Fig. 7H).

The binding site of mavacamten within the motor domain is observed to be identical between our cryo-EM independently determined structure and the bovine S1 crystal structure, yet distinct allosteric effects of mavacamten binding are seen within the cryo-EM derived structure, namely, pinching of the actin-binding cleft, and ADP.P_i_ and backdoor stabilization.

While these differences could be attributable to crystal packing effects or use of a bovine rather than a human construct, we suspect that these differences, seen specifically in the active site, are most likely attributable to the presence of the native ADP.P_i_ substrate within the cryo-EM structure and the analog ADP.BeFx in the crystal structure. ADP.BeFx is a strong inhibitor of myosin activity as it is a nonhydrolyzable analog of ATP.

Beryllium bonding is strictly tetrahedral and independent of the number of fluoride ions coordinating the Be atom, and the pentavalent trigonal bipyramidal geometry of the actual Pi transition state is excluded (*35*). This limits the conformations the analog can mimic within the active site and positions the BeFx closer to the ADP than a native P_i_ [discussed in further detail in (*35*) and observed in fig. S9]. This makes it impossible to interpret structural changes affecting P_i_ release when using such an analog; in the presence of mavacamten and ADP.BeFx, two inhibitory mechanisms are at play and the specific mechanism of mavacamten inhibition of P_i_ release cannot be deduced. Conversely, within the cryo-EM structure, the allosteric effects of mavacamten on ADP.P_i_ stabilization are observed, and structural evidence for the mechanism of inhibition of P_i_ release is provided.

With deeper insight into the impact of mavacamten on motor mechanics, future work can begin to apply this in a clinically meaningful setting. The exploration of HCM mutations in the mavacamten binding site and key interfaces created by mavacamten will provide insight into the functional effects of the drug in patients with specific mutations. For example, the HCM mutations R712L and E774V would directly affect the mavacamten binding site, likely rendering mavacamten less effective in patients with these mutations (*59*). The HCM mutations Arg^719^→Trp and Arg→^723^Gly, within immediate proximity of the binding site, do mildly affect mavacamten binding (*30*, *59*).

The newly formed network of motor-motor interactions in the presence of mavacamten also harbor several sites of pathogenic mutations: Asp^382^→Asn (*60*), Lys^383^→Val (*61*), Ala^393^→Val (*62*), Arg^719^→Trp (*63*), Arg^719^→Gln (*64*), Arg^719^→Pro (*65*), Gln^734^→Pro (*56*), and Gln^734^→Glu (*66*). These residues are not indicated to be directly involved in the motor-motor interface in the absence of mavacamten, based on the mavacamten-free IHM structure (PDB ID: 8ACT). So, these mutations may not directly destabilize the IHM, but they may affect myosin dynamics indirectly leading to less IHM formation. Thus, the IHM stabilizing effects of mavacamten in the presence of one of these mutations may be lessened.

Thus, further work is required to ascertain the effectiveness of mavacamten in the presence of disease-causing mutations within and surrounding the mavacamten binding site and at IHM contact interfaces. A deeper understanding of the mechanism through which mavacamten inhibits motor function in the context of disease may potentially allow us to predict patients who would be nonresponders or who would potentially suffer unintended side effects from treatment with mavacamten, such as a substantial reduction in systolic function. This would provide a path forward for personalized medicine alongside the development of more effective therapeutics.

## METHODS

### Adenovirus manipulation

The human β-cHMM used for cryo-EM encodes residues 1 to 1137 of the *MYH7* gene (GenBank: AAA51837.1). This design includes 42 heptad repeats of the S2 domain with a FLAG tag added at the C terminus (1138 to 1146) (fig. S3). A revised design was used for antibody capture for the motility assay that incorporates an epitope for an anti-S2 monoclonal antibody (mAb) (*37*, *67*). The antibody recognizes the epitope “AEKH RADLSRE” spanning heptads 43 and 44 of *MYH7*. The epitope was added into the coiled-coil S2 domain of β-cHMM followed by one additional heptad (45 heptads) and a FLAG tag at the C terminus. The single-headed cS1 design encodes residues 1 to 834 *MYH7* followed by a short linker fused to a green fluorescent protein (GFP) domain and a FLAG tag at the C terminus. The expression cassette includes two Internal Ribosome Entry Site (IRES) sequences for coexpression of human *MYL3* (vLC1) and *MYL2* (vLC2). The DNA sequences were assembled, inserted into an AdEasy shuttle vector, and sequenced (GeneWiz, Azenta Life Sciences, South Plainfield, NJ). Adenovirus plasmids were generated by recombination in *Escherichia coli* strain BJ5183-Ad1, and the transgene inserts in the plasmids were sequenced. New viruses were packaged and amplified in Ad293 cells through five passages to produce high titer (*68*). The virus was harvested and purified by CsCl density gradient sedimentation, yielding final virus titers of ~10^11^ infectious units per milliliter (IU ml^−1^) for infection of C2C12 cells and protein production.

### Muscle cell expression and purification of β-cHMM and S1

Maintenance of the mouse myogenic cell line, C2C12 (CRL 1772; American Type Culture Collection, Rockville, MD), has been described in detail elsewhere (*69*). Confluent C2C12 myoblasts were infected with replication defective recombinant adenovirus (AdcHMM2.0) at 2.7 × 10^8^ IU ml^−1^ in fusion medium (89% Dulbecco’s modified Eagle’s medium, 10% horse serum, and 1% fetal bovine serum). Expression of recombinant myosin (cHMM or cS1) was monitored by accumulation of coexpressed GFP fluorescence in infected cells. Myocyte differentiation and GFP accumulation were monitored until the cells were harvested (198 to 264 hours). Cells were chilled, medium was removed, and the cell layer was rinsed with cold phosphate-buffered saline (PBS). The cell layer was scraped into Triton extraction buffer: 100 mM NaCl, 0.5% Triton X-100, 10 mM imidazole (pH 7.0), 1 mM dithiothreitol (DTT), 5 mM MgATP, and protease inhibitor cocktail (Sigma-Aldrich, St. Louis). The cell suspension was collected in an ice-cold Dounce homogenizer and lysed with 15 strokes of the tight pestle. The cell debris in the whole cell lysate was pelleted by centrifugation at 17,000*g* for 15 min at 4°C. The Triton soluble extract was fractionated by ammonium sulfate precipitation using sequential steps of 0 to 30% saturation and 30 to 60% saturation. The myosin precipitates between 30 and 60% saturation of ammonium sulfate. The recovered pellet was dissolved in and dialyzed against 50 mM tris, 150 mM NaCl (pH 7.4), and 0.5 mM MgATP for affinity purification of the FLAG-tagged myosin on M2 mAb-Sepharose beads (Sigma-Aldrich). Bound myosin was eluted with FLAG peptide (0.1 mg ml^−1^; Sigma-Aldrich). Protein was concentrated and buffer was exchanged on Amicon Ultracel-10K centrifugal filters (Millipore, Darmstadt, Germany), dialyzed exhaustively into 10 mM Mops, 100 mM KCl, and 1 mM DTT before a final centrifugation at 300,000*g* for 10 min at 4°C. Aliquots were drop frozen in liquid nitrogen and stored in vapor phase at −147°C. The sequences of the β-cHMM and cS1 preparations used in this study were confirmed by liquid chromatography–tandem MS of protein digests. Bound light chains are those that are constitutively expressed in the C2C12 cells (MLC1/MLC3 and rLC2) or coexpressed with the cS1gfp (vLC1 and vLC2).

### In vitro motility assay

Measurement of in vitro motility of the S2 epitope–tagged cHMM was done as previously described (*37*, *70*). Nitrocellulose-coated glass coverslips were incubated with 0.1 mg/ml of the anti-S2 mAb, 10F12.3, in PBS followed by blocking the surface with 1% bovine serum albumin (BSA)/PBS. The cS1gfp was tethered with the anti-GFP mAb 3E6 (Invitrogen, Thermo Fisher Scientific) bound to nitrocellulose-coated glass coverslips. The β-cHMM and cS1 proteins were diluted to 20 μg/ml in motility buffer (MB) [25 mM imidazole (pH 7.8), 25 mM KCl, 4 mM MgCl_2_, 1 mM MgATP, and 1 mM DTT] supplemented with 1% BSA (MB/BSA). The antibody-coated coverslips were incubated with the proteins for 2 hours in a humidified chamber at 4°C. The coverslips were washed sequentially with MB/BSA and three times with MB before blocking with 0.5 μM unlabeled F-actin (5 min) and two final washes with MB. The coverslips were mounted on a 15-μl drop of 2 nM rhodamine-phalloidin–labeled actin in a modified MB [with 7.6 mM MgATP, 50 mM DTT, 0.5% methyl cellulose, glucose oxidase (0.1 mg/ml), catalase (0.018 mg/ml), and glucose (2.3 mg/ml)] in a small parafilm ring fixed on an alumina slide with vacuum grease. The chamber is observed with a temperature-controlled stage and the objective set at 32°C on an upright microscope with an image-intensified charge-coupled device (CCD) camera capturing images to an acquisition computer at 5 to 30 frames per second depending on assay parameters. Movement of actin filaments in two to three fields per slide for 500 frames per field of continuous imaging was captured and analyzed with semiautomated filament tracking programs as previously described (*71*). The trajectory of every filament with a lifetime of at least 10 frames is determined; the instantaneous velocity of the filament moving along the trajectory, the filament length, the distance of continuous motion, and the duration of pauses are tabulated. A weighted probability of the actin filament velocity for hundreds of events was fit to a Gaussian distribution and reported as the mean velocity and SD for each experimental condition.

### Negative stain head counting analysis

cHMM was first prepared by cross-linking with BS3 for 30 min at 25°C under the following conditions: 2 μM cHMM, 50 μM mavacamten (5% DMSO), 1 mM BS3, 1 mM EGTA, 1 mM ATP, 2 mM MgCl_2_, 56 mM KCl, and 10 mM Mops (pH 7.2). The reaction was then quenched with a 100 mM final concentration of tris (pH 8), preventing further cross-linking. Cross-linking was confirmed by SDS–polyacrylamide gel electrophoresis (SDS-PAGE) analysis (fig. S6).

Negative stain grids were prepared using the flicking method (*72*) by applying 5 μl of cross-linked cHMM diluted fivefold in buffer to a negative stain grid (produced in-house) glow-discharged for 30 s (PELCO easiGlow) prior to use. The excess HMM was then flicked off, and a drop of 2% uranyl acetate was applied. The excess was again flicked off, and the addition of a 2% uranyl acetate drop was repeated four times. Last, the grid was blotted with Whatman no. 42 ashless filter paper and dried. Negative stain image collection was performed using the FEI Tecan F20 equipped with an FEI CETA (CMOS CCD). Images were collected at 29,000× magnification at a defocus of −1 to −3 μm. Images were double blinded prior to counting of open and IHM-like particles. Images were only included in the analysis if a minimum of 250 particles were detected within the image such that the sample size was sufficient to obtain 80% power with a 95% confidence interval. Data were then plotted into box plots, and the significance in change between populations was calculated using a two-tailed Student’s *t* test in GraphPad Prism.

### Cryo-EM grid preparation and data collection

Prior to cryo-EM grid preparation, cHMM was cross-linked with BS3 using the same protocol as for negative stain with altered buffer conditions: 2 μM cHMM, 50 μM mavacamten (5% DMSO), 1 mM BS3, 1 mM EGTA, 1 mM ATP, 2 mM MgCl_2_, 50 mM KCl, and 10 mM Mops (pH 7). Grids were prepared using the Vitrobot Mark IV (Thermo Fisher Scientific). Three microliters of the BS3 cross-linked cHMM diluted twofold in buffer was applied to an UltrAufoil R 1.2/1.3 300-mesh gold grid (Quantifoil) for motor domain reconstruction and a UC-A on Lacey 400-mesh Cu continuous carbon grid (Agar Scientific) for IHM reconstruction, and glow-discharged for 30 s at 10 mA prior to use (PELCO easiGlow). These different grid types were chosen to optimize for visualization of each conformation, respectively. Thinner ice was obtained on gold grids, which largely occluded the IHM conformation, increasing the percentage of open head conformations observed within the cryo-EM images. Conversely, on the Lacey grid, an increase in the number of IHM particles was observed relative to open heads within the imageable areas. Grids were then blotted with Whatman no. 42 ashless filter paper (Agar Scientific, UK) for 1 s with a force of 6 and a wait time of 2 s at 8°C and 95% humidity before vitrification in liquid ethane. Data were collected on an FEI Titan Krios (Astbury Biostructure Laboratory, University of Leeds) operating at 300 kV equipped with a Falcon 4 direct electron camera with a specimen pixel size of 0.82 Å. Micrographs were collected using EPU acquisition software at 96,000× magnification with a total dose of 43 e^−^/Å^2^ and a defocus range of −1.2 to −3.0 μm. Total micrographs for each reconstruction were MD 9948 over one session, MD_mava_ 21,395 over two sessions, and IHM_mava_ 34,287 over three sessions.

### Cryo-EM data processing and model building

MD and MD_mava_ single motor domain image processing was carried out in RELION 4.0 (*73*) with subsequent processing in CryoSPARC v4.2 (*74*). Raw movies were imported into RELION for motion correction using MotionCor2 (*75*) and contrast transfer function (CTF) estimation using CTFFIND-4.1 (*76*). Automated particle picking was then performed using Topaz initially implementing the general model and then a trained Topaz (*77*) model on selected single motor domain two-dimensional (2D) classes; this was repeated for each dataset. Particles were extracted in a box size of 360^2^ pixels centered on individual motor domains. The resulting particles from both collections were then combined and classified using CryoSPARC reference-free 2D classification (*74*). Classes resembling single motor domains were selected for further classification. An initial model was generated using CryoSPARC’s ab initio reconstruction into five classes. The resulting maps were then refined through heterogeneous, homogeneous, and finally nonuniform refinement (*74*, *78*) on combined selected classes with a final particle stack of 88,809 MD and 200,487 MD_mava_. The resulting map was then sharpened using a negative B-factor automatically determined by CryoSPARC, and local resolution estimation was calculated in CryoSPARC (*74*). IHM_mava_ image processing followed the same pipeline, except that IHM 2D classes were selected to train a Topaz (*77*) model and only three ab initio (*74*) classes were used for initial model generation. A total of 197,869 particles contributed to the final map.

To interpret the cryo-EM maps, atomic models for the single motor domain and IHM were produced. Homology models for human βCM heavy and light chains were generated using the smooth muscle myosin shutdown structure (PDBID: 6Z47) (*79*). These models were then truncated at residue 796 (within the LCD) and flexibly fit to the single motor domain density using ISOLDE (*38*). Phenix real space refinement was then performed followed by adjustments in Coot (*80*) and ISOLDE (*38*); this was repeated until satisfied. The IHM model was then built using two MD_mava_ motor domain models joined to the homology modeled LCD, S2, and light chains. The model was flexibly fitted into the IHM map by use of ISOLDE and refined using Phenix real space refinement with final adjustments in Coot (*80*) and ISOLDE (*38*).

### qXL-MS sample preparation, measurement, data preparation, and analysis

Purified cS1 [25 μl; final concentration of 4 μM in 10 mM Mops (pH 7.3), 50 mM KCl, 1 mM MgCl_2_, 0.34 mM DTT, 1 mM EGTA, and 1 mM ATP] was incubated with 50 μM of mavacamten or DMSO control for 30 min at 25°C. DSBU (600 μM; 149 molar fold excess) was added, or DMSO control, and was allowed to react for 20 min at 25°C (final DMSO concentration of 1.6%, v/v). The reaction was quenched by the addition of tris (1 M, pH 7.3) to final concentration of 20 mM and incubation at room temperature for 15 min. Samples were flash frozen for storage prior to digestion. Cross-linking was confirmed by SDS-PAGE analysis (fig. S6).

Three replicates of both cross-linked and non–cross-linked control samples (≈14.5 μg) were processed for MS analysis using S-Trap micro spin columns (ProtiFi) as described previously (*81*). In brief, cross-linked samples were reduced by adding 20 mM DTT (10 min, 50°C) and then alkylated with 40 mM iodoacetamide (30 min, 20°C). The samples were acidified by the addition of phosphoric acid to a final concentration of 5% and subsequently diluted with 90% methanol in 100 mM triethylammonium bicarbonate (TEAB) (pH 7.1) (1:7, v/v, sample:buffer). The samples were then bound to an S-Trap micro spin column (ProtiFi). Subsequently, the column was washed three times with 90% methanol in 100 mM TEAB. A total of 1.44 μg of trypsin (Promega) was applied to the column, and digestion was then performed by incubating the S-Trap column at 47°C for 145 min. Peptides were recovered by washing the column sequentially with 50 mM TEAB (40 μl), 0.2% (v/v) formic acid (40 μl), and 50% acetonitrile/0.2% (v/v). The eluate was evaporated to dryness in a vacuum centrifuge, and the peptides were resuspended in 5% (v/v) acetonitrile/0.1% (v/v) formic acid (20 μl) prior to MS analysis.

Peptides, ≈10% of the final volume of each replicate, were analyzed on an Vanquish Neo LC (Thermo Fisher Scientific). Separation of peptides was performed using PepMap Neo C18 trap cartridge (Thermo Fisher Scientific, 174500) before using the EASY-Spray C18 column (Thermo Fisher Scientific, ES903). Elution of peptides from the column was achieved using a gradient elution of a 7.5 to 42.5% (v/v) solvent B (0.1%, v/v, formic acid in acetonitrile) in solvent A (0.1%, v/v, formic acid in water) over 97.5 min at 250 nl min^−1^. The eluate was infused into an Orbitrap Eclipse mass spectrometer (Thermo Fisher Scientific) operating in positive ion mode using an EASY-Spray nanoelectrospray ionization source.

An online exclusion list was generated from the MS1 measurement of a non–cross-linked control sample using the AcquireX AB workflow editor (Thermo Fisher Scientific application module) and was applied when performing MS analysis of the cross-linked samples. Data acquisition was performed in data-dependent analysis mode, and fragmentation was performed using collision dissociation activation at higher energy, as previously reported (*81*), but with use of an online exclusion list, generated from the MS1 measurement of a non–cross-linked control sample using the AcquireX AB workflow editor (Thermo Fisher Scientific application module).

The .RAW MS files produced were processed directly in FragPipe (v21.1) (*82*), without conversion to mzML, subject to a mass offset search using MSFragger (v4.0) (*82*) where default “Mass-Offset-Common-PTMs” workflow was loaded and amended. False discovery rate (FDR) at the protein/peptide/ion level was set to 1%, and tolerances for precursors/fragments were set to 10 parts per million (ppm). The generated calibrated mzML files were then taken forward as the generated interact.pep.xml file.

MeroX (v2.0.1.4) in RISEUP mode was used to search the replicate files individually for cross-links and monolinks (on residues K, S, T, and Y) against FASTA files for the cS1 construct and associated light chains as used for the MSFragger search, without the appended decoys and common contaminants from MSFragger. The search was performed with a 1.0% FDR and 50 score cutoff, precursor and fragment ion precision of 10 ppm with signal to noise ratio of 2.0, peptide length of 5 to 60 amino acids with up to five missed cleavage events, up to two methionine oxidation and two carbamidomethylation variable modifications, trimethylation of lysine as a posttranslational modification (PTM), variable N-terminal methionine clipping, monolinks reacting with water and tris, intrapeptide cross-link detection, and neutral loss of C_4_H_7_NO. In addition, three of the four DSBU reporter ion fragments needed to be present within the MS2 spectra to be included in the MeroX output.

The software package Skyline (*83*) (MacCoss Lab Software, v23.1.0.455) was used to quantify the cross-links and monolinks found by MeroX using a modified process laid out by Chen and Rappsilber (*41*) and since improved by Echeverri *et al.* (*84*) and separately by Jiang *et al.* (*85*). The six replicate MeroX search result .ZHRM files results were converted to the ProXL XML files as described by Riffle *et al.* (*86*). All cross-links and monolink peptides were manually curated, and all peaks of all transitions were manually inspected and aligned in Skyline to ensure that all three of the DMSO or mavacamten replicates had well defined MS1 peaks with matching retention times and indicative Skyline MS2 signal, with quantitation based on MS1 total ion count. In addition, as it has been our experience that MeroX can inconsistently report some cross-links and monolinks across multiple charge states or those bearing PTMs, we performed manual searches in Skyline and validation in SeeMS (*87*) when a non–DSBU-labeled peptide was identified under such conditions, yet concomitant cross-linked or monolink peptides were not identified in the MeroX search.

Cross- or monolinks within peptides where DSBU is potentially labeled on different residues, e.g., peptides bearing multiple K, S, T, and Y residues, which coelute and for which no clear determination of chromatographic peak assignment could be made, were manually inspected using SeeMS. Where geometric isomeric cross-linked or monolinked peptides exhibited chromatographic separation, MS2 spectra were inspected using SeeMS to assign the position of the cross-link using the reporter ion fragments. If no conclusive differentiation of the position of reactive residue could be made, then the MS1 signal was combined as if it was not chromatographically separated at baseline and the cross-link was assigned to the most reactive residue within the peptide (e.g., lysine, or where two lysines were present, the one that produced the most other cross-links or monolinks).

The signal intensity for each peptide for each replicate was normalized, as described by Chen and Rappsilber (*41*), using the total non–DSBU-reacted signal intensity of each replicate. The interact.pep.xml file from the MSFragger search was used to import the search of the six replicates into the Skyline file. Monolink signal intensity was combined from peptides that contain the same monolinked residue regardless of peptide charge state or length. Similarly, cross-link signal intensity was combined from dipeptides that bear the same residue to residue cross-link. An aligned interpeptide cross-link, intrapeptide cross-link, or monolink was determined to have changed significantly in the presence or absence of mavacamten by conducting pairwise comparisons via a single tailed, homoscedastic *t* test using relative signal intensity (data S2). The significance was measured by protein fold changes >2 and *P* < 0.05.

## References

[1] B. J. Maron, J. M. Gardin, J. M. Flack, S. S. Gidding, T. T. Kurosaki, D. E. Bild, Prevalence of hypertrophic cardiomyopathy in a general population of young adults. Circulation 92, 785–789 (1995). 7641357 10.1161/01.cir.92.4.785

[2] A. J. Marian, E. Braunwald, Hypertrophic cardiomyopathy. Circ. Res. 121, 749–770 (2017). 28912181 10.1161/CIRCRESAHA.117.311059 PMC5654557

[3] C. Y. Ho, Hypertrophic cardiomyopathy. Heart Fail. Clin. 6, 141–159 (2010). 20347784 10.1016/j.hfc.2009.12.001 PMC3031113

[4] M. Hamada, S. Ikeda, Y. Shigematsu, Advances in medical treatment of hypertrophic cardiomyopathy. J. Cardiol. 64, 1–10 (2014). 24735741 10.1016/j.jjcc.2014.02.022

[5] S. M. Day, J. C. Tardiff, E. M. Ostap, Myosin modulators: Emerging approaches for the treatment of cardiomyopathies and heart failure. J. Clin. Invest. 132, e148557 (2022). 35229734 10.1172/JCI148557 PMC8884898

[6] I. Olivotto, A. Oreziak, R. Barriales-Villa, T. P. Abraham, A. Masri, P. Garcia-Pavia, S. Saberi, N. K. Lakdawala, M. T. Wheeler, A. Owens, M. Kubanek, W. Wojakowski, M. K. Jensen, J. Gimeno-Blanes, K. Afshar, J. Myers, S. M. Hegde, S. D. Solomon, A. J. Sehnert, D. Zhang, W. Li, M. Bhattacharya, J. M. Edelberg, C. B. Waldman, S. J. Lester, A. Wang, C. Y. Ho, D. Jacoby, J. Bartunek, A. Bondue, E. Van Craenenbroeck, M. Kubanek, D. Zemanek, M. Jensen, J. Mogensen, J. J. Thune, P. Charron, A. Hagege, O. Lairez, J.-N. Trochu, C. Axthelm, H.-D. Duengen, N. Frey, V. Mitrovic, M. Preusch, J. Schulz-Menger, T. Seidler, M. Arad, M. Halabi, A. Katz, D. Monakier, O. Paz, S. Viskin, D. Zwas, I. Olivotto, H. P. Brunner-La Rocca, M. Michels, D. Dudek, Z. Oko-Sarnowska, A. Oreziak, W. Wojakowski, N. Cardim, H. Pereira, R. Barriales-Villa, P. García Pavia, J. Gimeno Blanes, R. Hidalgo Urbano, L. M. Rincón Diaz, P. Elliott, Z. Yousef, T. Abraham, K. Afshar, P. Alvarez, R. Bach, R. Becker, L. Choudhury, D. Fermin, D. Jacoby, J. Jefferies, C. Kramer, N. Lakdawala, S. Lester, A. Marian, A. Masri, M. Maurer, S. Nagueh, A. Owens, D. Owens, F. Rader, S. Saberi, M. Sherrid, J. Shirani, J. Symanski, A. Turer, A. Wang, O. Wever-Pinzon, M. Wheeler, T. Wong, M. Yamani, Mavacamten for treatment of symptomatic obstructive hypertrophic cardiomyopathy (EXPLORER-HCM): A randomised, double-blind, placebo-controlled, phase 3 trial. Lancet 396, 759–769 (2020). 32871100 10.1016/S0140-6736(20)31792-X

[7] E. M. Green, H. Wakimoto, R. L. Anderson, M. J. Evanchik, J. M. Gorham, B. C. Harrison, M. Henze, R. Kawas, J. D. Oslob, H. M. Rodriguez, Y. Song, W. Wan, L. A. Leinwand, J. A. Spudich, R. S. McDowell, J. G. Seidman, C. E. Seidman, A small-molecule inhibitor of sarcomere contractility suppresses hypertrophic cardiomyopathy in mice. Science 351, 617–621 (2016). 26912705 10.1126/science.aad3456 PMC4784435

[8] A. Amr, E. Kayvanpour, C. Reich, J. Koelemen, S. Asokan, N. Frey, B. Meder, F. Sedaghat-Hamedani, Assessing the applicability of cardiac myosin inhibitors for hypertrophic cardiomyopathy management in a large single center cohort. Rev. Cardiovasc. Med. 25, 225 (2024). 39076310 10.31083/j.rcm2506225 PMC11270100

[9] M. A. Sawan, S. Prabakaran, M. D’Souza, O. Behbahani-Nejad, M. E. Gold, B. R. Williams, O. Bilen, A systematic review of present and future pharmaco-structural therapies for hypertrophic cardiomyopathy. Clin. Cardiol. 47, e24207 (2024). 38269637 10.1002/clc.24207 PMC10766000

[10] L. Gorza, J. J. Mercadier, K. Schwartz, L. E. Thornell, S. Sartore, S. Schiaffino, Myosin types in the human heart. An immunofluorescence study of normal and hypertrophied atrial and ventricular myocardium. Circ. Res. 54, 694–702 (1984). 6234108 10.1161/01.res.54.6.694

[11] I. Rayment, W. R. Rypniewski, K. Schmidt-Bäse, R. Smith, D. R. Tomchick, M. M. Benning, D. A. Winkelmann, G. Wesenberg, H. M. Holden, Three-dimensional structure of myosin subfragment-1: A molecular motor. Science 261, 50–58 (1993). 8316857 10.1126/science.8316857

[12] J. Robert-Paganin, O. Pylypenko, C. Kikuti, H. L. Sweeney, A. Houdusse, Force generation by myosin motors: A structural perspective. Chem. Rev. 120, 5–35 (2020). 31689091 10.1021/acs.chemrev.9b00264

[13] D. Tamborrini, Z. Wang, T. Wagner, S. Tacke, M. Stabrin, M. Grange, A. L. Kho, M. Rees, P. Bennett, M. Gautel, S. Raunser, Structure of the native myosin filament in the relaxed cardiac sarcomere. Nature 623, 863–871 (2023). 37914933 10.1038/s41586-023-06690-5 PMC10665186

[14] D. Dutta, V. Nguyen, K. S. Campbell, R. Padron, R. Craig, Cryo-EM structure of the human cardiac myosin filament. Nature 623, 853–862 (2023). 37914935 10.1038/s41586-023-06691-4 PMC10846670

[15] P. B. Conibear, C. R. Bagshaw, P. G. Fajer, M. Kovács, A. Málnási-Csizmadia, Myosin cleft movement and its coupling to actomyosin dissociation. Nat. Struct. Biol. 10, 831–835 (2003). 14502269 10.1038/nsb986

[16] M. A. Geeves, K. C. Holmes, The molecular mechanism of muscle contraction. Adv. Protein Chem. 71, 161–193 (2005). 16230112 10.1016/S0065-3233(04)71005-0

[17] M. A. Geeves, K. C. Holmes, Structural mechanism of muscle contraction. Annu. Rev. Biochem. 68, 687–728 (1999). 10872464 10.1146/annurev.biochem.68.1.687

[18] D. P. Klebl, S. N. McMillan, C. Risi, E. Forgacs, B. Virok, J. L. Atherton, S. A. Harris, M. Stofella, D. A. Winkelmann, F. Sobott, V. E. Galkin, P. J. Knight, S. P. Muench, C. A. Scarff, H. D. White, Swinging lever mechanism of myosin directly shown by time-resolved cryoEM. Nature 642, 519–526 (2025). 40205053 10.1038/s41586-025-08876-5 PMC12158783

[19] M. H. Doran, M. J. Rynkiewicz, D. Rasicci, S. M. L. Bodt, M. E. Barry, E. Bullitt, C. M. Yengo, J. R. Moore, W. Lehman, Conformational changes linked to ADP release from human cardiac myosin bound to actin-tropomyosin. J. Gen. Physiol. 155, e202213267 (2023). 36633586 10.1085/jgp.202213267 PMC9859928

[20] R. W. Lymn, E. W. Taylor, Mechanism of adenosine triphosphate hydrolysis by actomyosin. Biochemistry 10, 4617–4624 (1971). 4258719 10.1021/bi00801a004

[21] S. K. Barrick, M. J. Greenberg, Cardiac myosin contraction and mechanotransduction in health and disease. J. Biol. Chem. 297, 101297 (2021). 34634306 10.1016/j.jbc.2021.101297 PMC8559575

[22] M. Irving, Functional control of myosin motors in the cardiac cycle. Nat. Rev. Cardiol. 22, 9–19 (2025). 39030271 10.1038/s41569-024-01063-5

[23] L. S. Tobacman, Thin filament-mediated regulation of cardiac contraction. Annu. Rev. Physiol. 58, 447–481 (1996). 8815803 10.1146/annurev.ph.58.030196.002311

[24] E. Brunello, L. Fusi, A. Ghisleni, S. J. Park-Holohan, J. G. Ovejero, T. Narayanan, M. Irving, Myosin filament-based regulation of the dynamics of contraction in heart muscle. Proc. Natl. Acad. Sci. U.S.A. 117, 8177–8186 (2020). 32220962 10.1073/pnas.1920632117 PMC7149498

[25] J. A. Spudich, Three perspectives on the molecular basis of hypercontractility caused by hypertrophic cardiomyopathy mutations. Pflügers Arch. 471, 701–717 (2019). 30767072 10.1007/s00424-019-02259-2 PMC6475635

[26] S. S. Sarkar, D. V. Trivedi, M. M. Morck, A. S. Adhikari, S. N. Pasha, K. M. Ruppel, J. A. Spudich, The hypertrophic cardiomyopathy mutations R403Q and R663H increase the number of myosin heads available to interact with actin. Sci. Adv. 6, eaax0069 (2020). 32284968 10.1126/sciadv.aax0069 PMC7124958

[27] A. S. Adhikari, K. B. Kooiker, S. S. Sarkar, C. Liu, D. Bernstein, J. A. Spudich, K. M. Ruppel, Early-onset hypertrophic cardiomyopathy mutations significantly increase the velocity, force, and actin-activated ATPase activity of human β-cardiac myosin. Cell Rep. 17, 2857–2864 (2016). 27974200 10.1016/j.celrep.2016.11.040 PMC11088367

[28] J. A. Spudich, N. Nandwani, J. Robert-Paganin, A. Houdusse, K. M. Ruppel, Reassessing the unifying hypothesis for hypercontractility caused by myosin mutations in hypertrophic cardiomyopathy. EMBO J. 43, 4139–4155 (2024). 39192034 10.1038/s44318-024-00199-x PMC11445530

[29] B. J. Maron, M. Y. Desai, R. A. Nishimura, P. Spirito, H. Rakowski, J. A. Towbin, E. J. Rowin, M. S. Maron, M. V. Sherrid, Diagnosis and evaluation of hypertrophic cardiomyopathy. J. Am. Coll. Cardiol. 79, 372–389 (2022). 35086660 10.1016/j.jacc.2021.12.002

[30] R. F. Kawas, R. L. Anderson, S. R. B. Ingle, Y. Song, A. S. Sran, H. M. Rodriguez, A small-molecule modulator of cardiac myosin acts on multiple stages of the myosin chemomechanical cycle. J. Biol. Chem. 292, 16571–16577 (2017). 28808052 10.1074/jbc.M117.776815 PMC5633120

[31] J. A. Rohde, O. Roopnarine, D. D. Thomas, J. M. Muretta, Mavacamten stabilizes an autoinhibited state of two-headed cardiac myosin. Proc. Natl. Acad. Sci. U.S.A. 115, E7486–E7494 (2018). 30018063 10.1073/pnas.1720342115 PMC6094135

[32] S. Nag, S. K. Gollapudi, C. L. del Rio, J. A. Spudich, R. McDowell, Mavacamten, a precision medicine for hypertrophic cardiomyopathy: From a motor protein to patients. Sci. Adv. 9, eabo7622 (2023). 37506209 10.1126/sciadv.abo7622 PMC12494088

[33] L. Chen, J. Liu, H. Rastegarpouyani, P. M. L. Janssen, J. R. Pinto, K. A. Taylor, Structure of mavacamten-free human cardiac thick filaments within the sarcomere by cryoelectron tomography. Proc. Natl. Acad. Sci. U.S.A. 121, e2311883121 (2024). 38386705 10.1073/pnas.2311883121 PMC10907299

[34] D. Auguin, J. Robert-Paganin, S. Réty, C. Kikuti, A. David, G. Theumer, A. W. Schmidt, H.-J. Knölker, A. Houdusse, Omecamtiv mecarbil and mavacamten target the same myosin pocket despite opposite effects in heart contraction. Nat. Commun. 15, 4885 (2024). 38849353 10.1038/s41467-024-47587-9 PMC11161628

[35] M. Chabre, Aluminofluoride and beryllofluoride complexes: New phosphate analogs in enzymology. Trends Biochem. Sci. 15, 6–10 (1990). 2180149 10.1016/0968-0004(90)90117-t

[36] C. Toepfer, J. R. Sellers, Use of fluorescent techniques to study the in vitro movement of myosins. Exp. Suppl. 105, 193–210 (2014). 25095996 10.1007/978-3-0348-0856-9_9 PMC4178934

[37] B. Barua, R. C. Cail, Y. E. Goldman, E. M. Ostap, D. A. Winkelmann, S2Tag, a novel affinity tag for the capture and immobilization of coiled-coil proteins: Application to the study of human β-cardiac myosin. J. Biol. Chem. 301, 110776 (2025). 41033555 10.1016/j.jbc.2025.110776 PMC12597268

[38] T. I. Croll, ISOLDE: A physically realistic environment for model building into low-resolution electron-density maps. Acta Crystallogr. D. Struct. Biol. 74, 519–530 (2018). 29872003 10.1107/S2059798318002425 PMC6096486

[39] R. L. Anderson, D. V. Trivedi, S. S. Sarkar, M. Henze, W. Ma, H. Gong, C. S. Rogers, J. M. Gorham, F. L. Wong, M. M. Morck, J. G. Seidman, K. M. Ruppel, T. C. Rving, R. Cooke, E. M. Green, J. A. Spudich, Deciphering the super relaxed state of human β-cardiac myosin and the mode of action of mavacamten from myosin molecules to muscle fibers. Proc. Natl. Acad. Sci. U.S.A. 115, E8143–E8152 (2018). 30104387 10.1073/pnas.1809540115 PMC6126717

[40] F. Müller, L. Fischer, Z. A. Chen, T. Auchynnikava, J. Rappsilber, On the reproducibility of label-free quantitative cross-linking/mass spectrometry. J. Am. Soc. Mass Spectrom. 29, 405–412 (2018). 29256016 10.1007/s13361-017-1837-2 PMC5814520

[41] Z. A. Chen, J. Rappsilber, Quantitative cross-linking/mass spectrometry to elucidate structural changes in proteins and their complexes. Nat. Protoc. 14, 171–201 (2019). 30559374 10.1038/s41596-018-0089-3

[42] C. Iacobucci, M. Götze, C. H. Ihling, C. Piotrowski, C. Arlt, M. Schäfer, C. Hage, R. Schmidt, A. Sinz, A cross-linking/mass spectrometry workflow based on MS-cleavable cross-linkers and the MeroX software for studying protein structures and protein-protein interactions. Nat. Protoc. 13, 2864–2889 (2018). 30382245 10.1038/s41596-018-0068-8

[43] E. D. Merkley, S. Rysavy, A. Kahraman, R. P. Hafen, V. Daggett, J. N. Adkins, Distance restraints from crosslinking mass spectrometry: Mining a molecular dynamics simulation database to evaluate lysine–lysine distances. Protein Sci. 23, 747–759 (2014). 24639379 10.1002/pro.2458 PMC4093951

[44] A. Grinzato, D. Auguin, C. Kikuti, N. Nandwani, D. Moussaoui, D. Pathak, E. Kandiah, K. M. Ruppel, J. A. Spudich, A. Houdusse, J. Robert-Paganin, Cryo-EM structure of the folded-back state of human β-cardiac myosin. Nat. Commun. 14, 3166 (2023). 37258552 10.1038/s41467-023-38698-w PMC10232470

[45] H. Watkins, A. Rosenzweig, D. S. Hwang, T. Levi, W. McKenna, C. E. Seidman, J. G. Seidman, Characteristics and prognostic implications of myosin missense mutations in familial hypertrophic cardiomyopathy. N. Engl. J. Med. 326, 1108–1114 (1992). 1552912 10.1056/NEJM199204233261703

[46] B. Yu, N. A. Sawyer, M. Caramins, Z. G. Yuan, R. B. Saunderson, R. Pamphlett, D. R. Richmond, R. W. Jeremy, R. J. Trent, Denaturing high performance liquid chromatography: High throughput mutation screening in familial hypertrophic cardiomyopathy and SNP genotyping in motor neurone disease. J. Clin. Pathol. 58, 479–485 (2005). 15858117 10.1136/jcp.2004.021642 PMC1770671

[47] A. Frazier, D. P. Judge, S. P. Schulman, N. Johnson, K. W. Holmes, A. M. Murphy, Familial hypertrophic cardiomyopathy associated with cardiac β-myosin heavy chain and troponin I mutations. Pediatr. Cardiol. 29, 846–850 (2008). 18175163 10.1007/s00246-007-9177-9

[48] P. D. Stenson, E. V. Ball, M. Mort, A. D. Phillips, J. A. Shiel, N. S. Thomas, S. Abeysinghe, M. Krawczak, D. N. Cooper, Human gene mutation database (HGMD): 2003 Update. Hum. Mutat. 21, 577–581 (2003). 12754702 10.1002/humu.10212

[49] K. Curila, L. Benesova, M. Penicka, M. Minarik, D. Zemanek, J. Veselka, P. Widimsky, P. Gregor, Spectrum and clinical manifestations of mutations in genes responsible for hypertrophic cardiomyopathy. Acta Cardiol. 67, 23–29 (2012). 22455086 10.1080/ac.67.1.2146562

[50] C. Y. Ho, N. K. Sweitzer, B. McDonough, B. J. Maron, S. A. Casey, J. G. Seidman, C. E. Seidman, S. D. Solomon, Assessment of diastolic function with Doppler tissue imaging to predict genotype in preclinical hypertrophic cardiomyopathy. Circulation 105, 2992–2997 (2002). 12081993 10.1161/01.cir.0000019070.70491.6d

[51] S. L. Van Driest, M. A. Jaeger, S. R. Ommen, M. L. Will, B. J. Gersh, A. J. Tajik, M. J. Ackerman, Comprehensive analysis of the beta-myosin heavy chain gene in 389 unrelated patients with hypertrophic cardiomyopathy. J. Am. Coll. Cardiol. 44, 602–610 (2004). 15358028 10.1016/j.jacc.2004.04.039

[52] H. Morita, H. L. Rehm, A. Menesses, B. McDonough, A. E. Roberts, R. Kucherlapati, J. A. Towbin, J. G. Seidman, C. E. Seidman, Shared genetic causes of cardiac hypertrophy in children and adults. N. Engl. J. Med. 358, 1899–1908 (2008). 18403758 10.1056/NEJMoa075463 PMC2752150

[53] I. Olivotto, F. Girolami, M. J. Ackerman, S. Nistri, J. M. Bos, E. Zachara, S. R. Ommen, J. L. Theis, R. A. Vaubel, F. Re, C. Armentano, C. Poggesi, F. Torricelli, F. Cecchi, Myofilament protein gene mutation screening and outcome of patients with hypertrophic cardiomyopathy. Mayo Clin. Proc. 83, 630–638 (2008). 18533079 10.4065/83.6.630

[54] K. E. Berge, T. P. Leren, Genetics of hypertrophic cardiomyopathy in Norway. Clin. Genet. 86, 355–360 (2014). 24111713 10.1111/cge.12286

[55] E. Arbustini, R. Fasani, P. Morbini, M. Diegoli, M. Grasso, B. Dal Bello, E. Marangoni, P. Banfi, N. Banchieri, O. Bellini, G. Comi, J. Narula, C. Campana, A. Gavazzi, C. Danesino, M. Viganò, Coexistence of mitochondrial DNA and beta myosin heavy chain mutations in hypertrophic cardiomyopathy with late congestive heart failure. Heart 80, 548–558 (1998). 10065021 10.1136/hrt.80.6.548 PMC1728869

[56] L. Song, Y. Zou, J. Wang, Z. Wang, Y. Zhen, K. Lou, Q. Zhang, X. Wang, H. Wang, J. Li, R. Hui, Mutations profile in Chinese patients with hypertrophic cardiomyopathy. Clin. Chim. Acta 351, 209–216 (2005). 15563892 10.1016/j.cccn.2004.09.016

[57] S. A. Mohiddin, D. A. Begley, E. McLam, J. P. Cardoso, J. B. Winkler, J. R. Sellers, L. Fananapazir, Utility of genetic screening in hypertrophic cardiomyopathy: Prevalence and significance of novel and double (homozygous and heterozygous) β-myosin mutations. Genet. Test. 7, 21–27 (2003). 12820698 10.1089/109065703321560895

[58] Y. Zou, J. Wang, X. Liu, Y. Wang, Y. Chen, K. Sun, S. Gao, C. Zhang, Z. Wang, Y. Zhang, X. Feng, Y. Song, Y. Wu, H. Zhang, L. Jia, H. Wang, D. Wang, C. Yan, M. Lu, X. Zhu, L. Song, R. Hui, Multiple gene mutations, not the type of mutation, are the modifier of left ventricle hypertrophy in patients with hypertrophic cardiomyopathy. Mol. Biol. Rep. 40, 3969–3976 (2013). 23283745 10.1007/s11033-012-2474-2

[59] A. Snoberger, B. Barua, J. L. Atherton, H. Shuman, E. Forgacs, Y. E. Goldman, D. A. Winkelmann, E. M. Ostap, Myosin with hypertrophic cardiac mutation R712L has a decreased working stroke which is rescued by omecamtiv mecarbil. eLife 10, e63691 (2021). 33605878 10.7554/eLife.63691 PMC7895523

[60] J. P. Kaski, P. Syrris, M. T. T. Esteban, S. Jenkins, A. Pantazis, J. E. Deanfield, W. J. McKenna, P. M. Elliott, Prevalence of sarcomere protein gene mutations in preadolescent children with hypertrophic cardiomyopathy. Circ. Cardiovasc. Genet. 2, 436–441 (2009). 20031618 10.1161/CIRCGENETICS.108.821314

[61] S. Q. Kuang, J. D. Yu, L. Lu, L. M. He, L. S. Gong, S. J. Chen, Z. Chen, Identification of a novel missense mutation in the cardiac β-myosin heavy chain gene in a Chinese patient with sporadic hypertrophic cardiomyopathy. J. Mol. Cell. Cardiol. 28, 1879–1883 (1996). 8899546 10.1006/jmcc.1996.0180

[62] O. R. Mook, M. A. Haagmans, J. F. Soucy, J. B. van de Meerakker, F. Baas, M. E. Jakobs, N. Hofman, I. Christiaans, R. H. Lekanne Deprez, M. M. Mannens, Targeted sequence capture and GS-FLX Titanium sequencing of 23 hypertrophic and dilated cardiomyopathy genes: Implementation into diagnostics. J. Med. Genet. 50, 614–626 (2013). 23785128 10.1136/jmedgenet-2012-101231 PMC3756457

[63] R. Anan, G. Greve, L. Thierfelder, H. Watkins, W. J. McKenna, S. Solomon, C. Vecchio, H. Shono, S. Nakao, H. Tanaka, Prognostic implications of novel beta cardiac myosin heavy chain gene mutations that cause familial hypertrophic cardiomyopathy. J. Clin. Invest. 93, 280–285 (1994). 8282798 10.1172/JCI116957 PMC293763

[64] M. W. Consevage, G. C. Salada, B. G. Baylen, R. L. Ladda, P. K. Rogan, A new missense mutation, Arg719Gln, in the β-cardiac heavy chain myosin gene of patients with familial hypertrophic cardiomyopathy. Hum. Mol. Genet. 3, 1025–1026 (1994). 7848441 10.1093/hmg/3.6.1025

[65] M. García-Castro, E. Coto, J. R. Reguero, J. R. Berrazueta, V. Alvarez, B. Alonso, R. Sainz, M. Martín, C. Morís, Mutations in sarcomeric genes MYH7, MYBPC3, TNNT2, TNNI3, and TPM1 in patients with hypertrophic cardiomyopathy. Rev. Esp. Cardiol. 62, 48–56 (2009). 19150014

[66] L. Nanni, M. Pieroni, C. Chimenti, B. Simionati, R. Zimbello, A. Maseri, A. Frustaci, G. Lanfranchi, Hypertrophic cardiomyopathy: Two homozygous cases with “typical” hypertrophic cardiomyopathy and three new mutations in cases with progression to dilated cardiomyopathy. Biochem. Biophys. Res. Commun. 309, 391–398 (2003). 12951062 10.1016/j.bbrc.2003.08.014

[67] D. A. Winkelmann, L. Bourdieu, A. Ott, F. Kinose, A. Libchaber, Flexibility of myosin attachment to surfaces influences F-actin motion. Biophys. J. 68, 2444–2453 (1995). 7544167 10.1016/S0006-3495(95)80426-1 PMC1282154

[68] J. Luo, Z. L. Deng, X. Luo, N. Tang, W. X. Song, J. Chen, K. A. Sharff, H. H. Luu, R. C. Haydon, K. W. Kinzler, B. Vogelstein, T. C. He, A protocol for rapid generation of recombinant adenoviruses using the AdEasy system. Nat. Protoc. 2, 1236–1247 (2007). 17546019 10.1038/nprot.2007.135

[69] Q. Wang, C. L. Moncman, D. A. Winkelmann, Mutations in the motor domain modulate myosin activity and myofibril organization. J. Cell Sci. 116, 4227–4238 (2003). 12953063 10.1242/jcs.00709

[70] R. C. Cail, B. Barua, F. A. Báez-Cruz, D. A. Winkelmann, Y. E. Goldman, E. M. Ostap, A myosin hypertrophic cardiomyopathy mutation disrupts the super-relaxed state and boosts contractility by enhanced actin attachment. Proc. Natl. Acad. Sci. U.S.A. 122, e2521561122 (2025). 41439707 10.1073/pnas.2521561122 PMC12772213

[71] B. Barua, D. A. Winkelmann, H. D. White, S. E. Hitchcock-DeGregori, Regulation of actin-myosin interaction by conserved periodic sites of tropomyosin. Proc. Natl. Acad. Sci. U.S.A. 109, 18425–18430 (2012). 23091026 10.1073/pnas.1212754109 PMC3494946

[72] C. A. Scarff, M. J. G. Fuller, R. F. Thompson, M. G. Iadanza, Variations on negative stain electron microscopy methods: Tools for tackling challenging systems. J. Vis. Exp., 57199 (2018). 29443097 10.3791/57199 PMC5912373

[73] D. Kimanius, L. Dong, G. Sharov, T. Nakane, S. H. W. Scheres, New tools for automated cryo-EM single-particle analysis in RELION-4.0. Biochem. J. 478, 4169–4185 (2021). 34783343 10.1042/BCJ20210708 PMC8786306

[74] A. Punjani, J. L. Rubinstein, D. J. Fleet, M. A. Brubaker, cryoSPARC: Algorithms for rapid unsupervised cryo-EM structure determination. Nat. Methods 14, 290–296 (2017). 28165473 10.1038/nmeth.4169

[75] S. Q. Zheng, E. Palovcak, J. P. Armache, K. A. Verba, Y. Cheng, D. A. Agard, MotionCor2: Anisotropic correction of beam-induced motion for improved cryo-electron microscopy. Nat. Methods 14, 331–332 (2017). 28250466 10.1038/nmeth.4193 PMC5494038

[76] A. Rohou, N. Grigorieff, CTFFIND4: Fast and accurate defocus estimation from electron micrographs. J. Struct. Biol. 192, 216–221 (2015). 26278980 10.1016/j.jsb.2015.08.008 PMC6760662

[77] T. Bepler, A. Morin, M. Rapp, J. Brasch, L. Shapiro, A. J. Noble, B. Berger, Positive-unlabeled convolutional neural networks for particle picking in cryo-electron micrographs. Nat. Methods 16, 1153–1160 (2019). 31591578 10.1038/s41592-019-0575-8 PMC6858545

[78] A. Punjani, H. Zhang, D. J. Fleet, Non-uniform refinement: Adaptive regularization improves single-particle cryo-EM reconstruction. Nat. Methods 17, 1214–1221 (2020). 33257830 10.1038/s41592-020-00990-8

[79] C. A. Scarff, G. Carrington, D. Casas-Mao, J. M. Chalovich, P. J. Knight, N. A. Ranson, M. Peckham, Structure of the shutdown state of myosin-2. Nature 588, 515–520 (2020). 33268888 10.1038/s41586-020-2990-5 PMC7611489

[80] P. Emsley, B. Lohkamp, W. G. Scott, K. Cowtan, Features and development of Coot. Acta Crystallogr. D Biol. Crystallogr. 66, 486–501 (2010). 20383002 10.1107/S0907444910007493 PMC2852313

[81] L. Makhlouf, J. J. Peter, H. M. Magnussen, R. Thakur, D. Millrine, T. C. Minshull, G. Harrison, J. Varghese, F. Lamoliatte, M. Foglizzo, T. Macartney, A. N. Calabrese, E. Zeqiraj, Y. Kulathu, The UFM1 E3 ligase recognizes and releases 60S ribosomes from ER translocons. Nature 627, 437–444 (2024). 38383789 10.1038/s41586-024-07093-w PMC10937380

[82] A. T. Kong, F. V. Leprevost, D. M. Avtonomov, D. Mellacheruvu, A. I. Nesvizhskii, MSFragger: Ultrafast and comprehensive peptide identification in mass spectrometry–based proteomics. Nat. Methods 14, 513–520 (2017). 28394336 10.1038/nmeth.4256 PMC5409104

[83] L. K. Pino, B. C. Searle, J. G. Bollinger, B. Nunn, B. MacLean, M. J. MacCoss, The Skyline ecosystem: Informatics for quantitative mass spectrometry proteomics. Mass Spectrom. Rev. 39, 229–244 (2020). 28691345 10.1002/mas.21540 PMC5799042

[84] J. C. Rojas Echeverri, F. Hause, C. Iacobucci, C. H. Ihling, D. Tänzler, N. Shulman, M. Riffle, B. X. MacLean, A. Sinz, A workflow for improved analysis of cross-linking mass spectrometry data integrating parallel accumulation-serial fragmentation with MeroX and skyline. Anal. Chem. 96, 7373–7379 (2024). 38696819 10.1021/acs.analchem.4c00829 PMC11099889

[85] T. Jiang, G. Wan, H. Zhang, Y. P. Gyawali, E. S. Underbakke, C. Feng, Probing protein dynamics in neuronal nitric oxide synthase by quantitative cross-linking mass spectrometry. Biochemistry 62, 2232–2237 (2023). 37459398 10.1021/acs.biochem.3c00245 PMC10529231

[86] M. Riffle, D. Jaschob, A. Zelter, T. N. Davis, ProXL (protein cross-linking database): A platform for analysis, visualization, and sharing of protein cross-linking mass spectrometry data. J. Proteome Res. 15, 2863–2870 (2016). 27302480 10.1021/acs.jproteome.6b00274 PMC4977572

[87] M. C. Chambers, B. Maclean, R. Burke, D. Amodei, D. L. Ruderman, S. Neumann, L. Gatto, B. Fischer, B. Pratt, J. Egertson, K. Hoff, D. Kessner, N. Tasman, N. Shulman, B. Frewen, T. A. Baker, M. Y. Brusniak, C. Paulse, D. Creasy, L. Flashner, K. Kani, C. Moulding, S. L. Seymour, L. M. Nuwaysir, B. Lefebvre, F. Kuhlmann, J. Roark, P. Rainer, S. Detlev, T. Hemenway, A. Huhmer, J. Langridge, B. Connolly, T. Chadick, K. Holly, J. Eckels, E. W. Deutsch, R. L. Moritz, J. E. Katz, D. B. Agus, M. MacCoss, D. L. Tabb, P. Mallick, A cross-platform toolkit for mass spectrometry and proteomics. Nat. Biotechnol. 30, 918–920 (2012). 23051804 10.1038/nbt.2377 PMC3471674

[88] Y. Perez-Riverol, J. Bai, C. Bandla, D. García-Seisdedos, S. Hewapathirana, S. Kamatchinathan, D. J. Kundu, A. Prakash, A. Frericks-Zipper, M. Eisenacher, M. Walzer, S. Wang, A. Brazma, J. A. Vizcaíno, The PRIDE database resources in 2022: A hub for mass spectrometry-based proteomics evidences. Nucleic Acids Res. 50, D543–D552 (2022). 34723319 10.1093/nar/gkab1038 PMC8728295

