# Supplementary material for: Mavacamten inhibits myosin activity by stabilizing the myosin interacting-heads motif and stalling motor force generation
Source: Sci Adv. 2026 Apr 29;12(18):eaea9335. doi: 10.1126/sciadv.aea9335 (PMC13127578; doi:10.1126/sciadv.aea9335)
Supplement: Supplementary file 1 — Figs. S1 to S15 Tables S1 to S5 Legends for movies S1 to S3 Legends for data S1 and S2 [file sciadv.aea9335_sm.pdf]

Science Advances
AAAS

Supplementary Materials for

Mavacamten inhibits myosin activity by stabilizing the myosin interacting-heads motif and stalling motor force generation

Sean N. McMillan *et al.*

Corresponding author: Charlotte A. Scarff, c.a.scarff@leeds.ac.uk

Sci. Adv. **12**, eaea9335 (2026)
DOI: 10.1126/sciadv.aea9335

The PDF file includes:

Figs. S1 to S15
Tables S1 to S5
Legends for movies S1 to S3
Legends for data S1 and S2

Other Supplementary Material for this manuscript includes the following:

Movies S1 to S3
Data S1 and S2

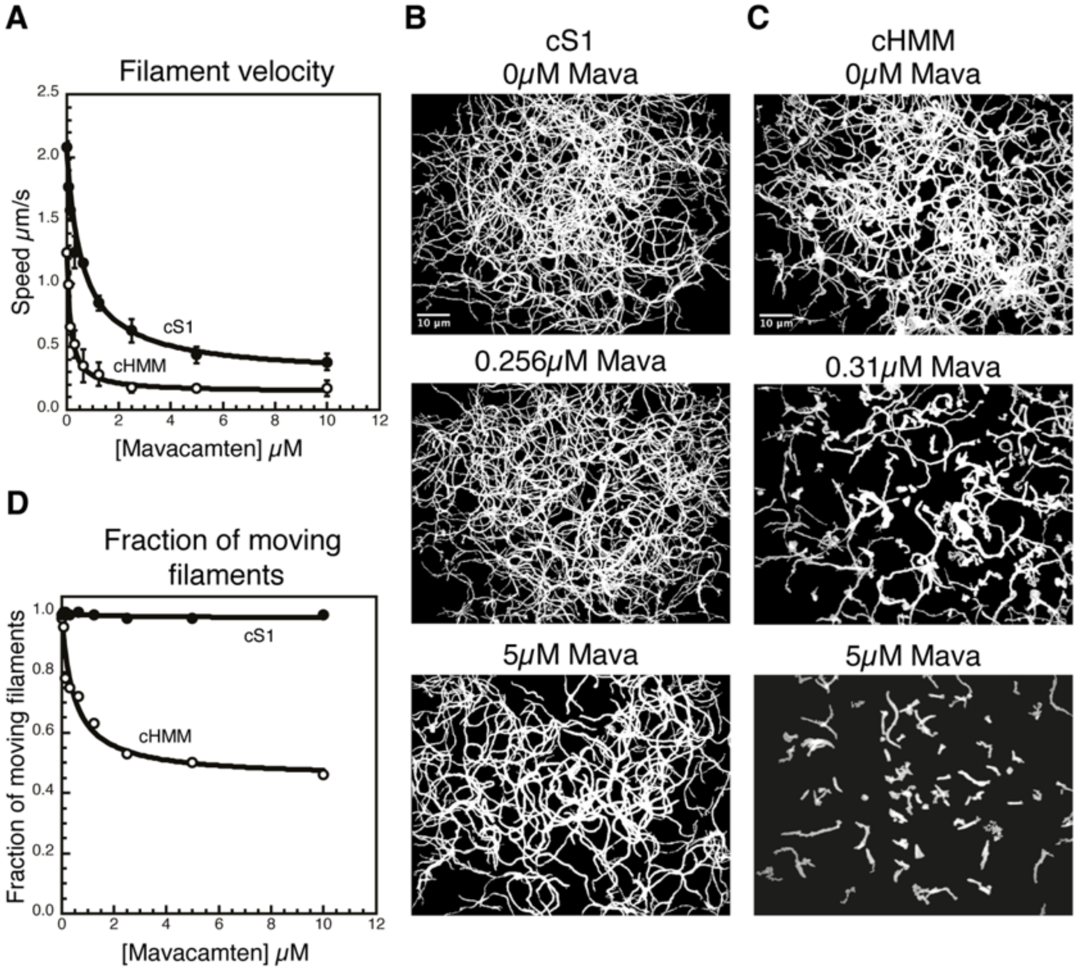

Fig. S1. Mavacamten inhibits the gliding velocity of actin filaments more effectively for cHMM compared to cS1. (a) Actin filament gliding velocity for cS1 and cHMM over a titration of mavacamten. The filament speed powered by cS1 is slowed up to 86 % by mavacamten with an IC_{50} of 0.62 μM. Comparatively, the filament speed powered by cHMM is slowed by 90 % with a 4-fold lower IC_{50} of 0.14 μM. (b-c) Summed plot of actin filament movement over 100 seconds of motility for (b) cS1 and (c) cHMM at mavacamten concentrations of 0 μM, ~0.3 μM and 5 μM. (d) Analysis of the number of moving filaments for cS1 and cHMM over a titration of mavacamten. Mavacamten has no impact on the fraction of filaments moving for cS1 however, mavacamten decreases the fraction of moving filaments for cHMM in a concentration dependent manner.

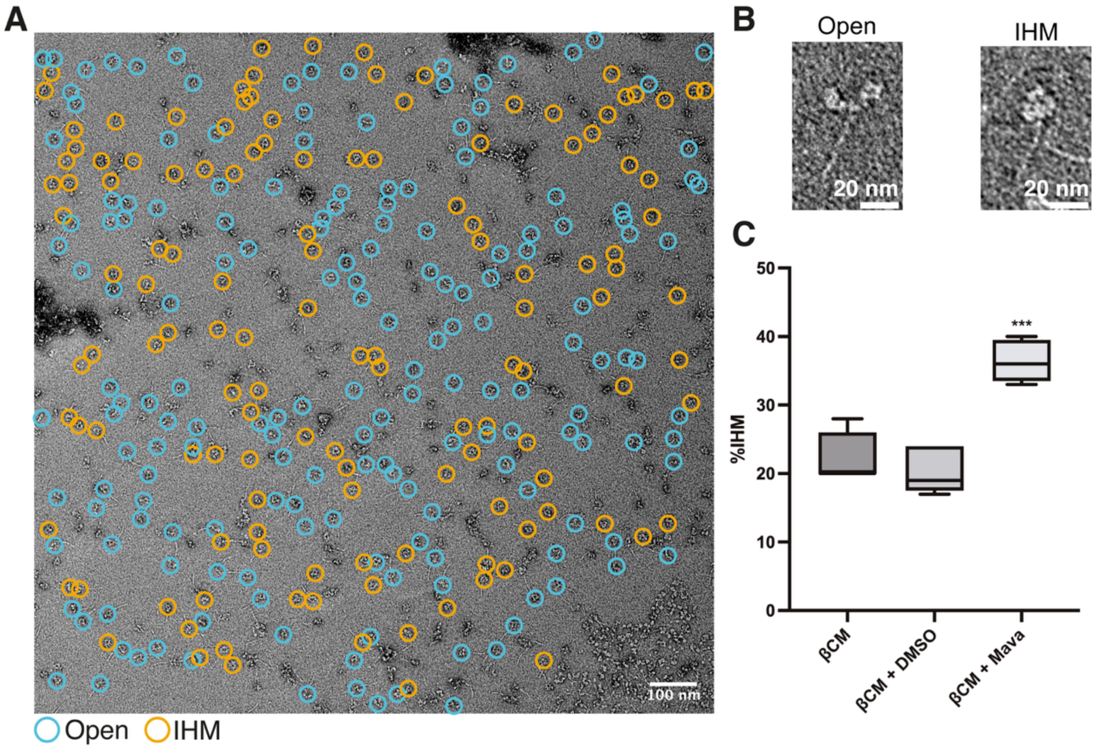

Fig. S2. Direct observation of IHM stabilisation due to mavacamten. (A) Representative negative-stain EM micrograph used during head counting assay. Counted bCM particles highlighted by coloured circle, blue: open, orange: IHM. (b) Representative negative stain open and IHM bCM molecule. (c) Box plot showing percentage of IHM over 5 biological replicates. The bCM control resulted in a median of 20 % with a first and 3rd quartile of 20 % and 24 % respectively. bCM containing 2.5 % v/v DMSO showed a median of 19 % with a first and third quartile of 18 % and 24 % with no significant difference to the bCM control determined by an unpaired two-tailed student t-test. Mavacamten shows a median of 36 % with a first and third quartile of 34 % and 39 %, a significant increase in % IHM formation with a P-value of = 0.0002 determined by an unpaired two-tailed student t-test with respect to the ßCM control.

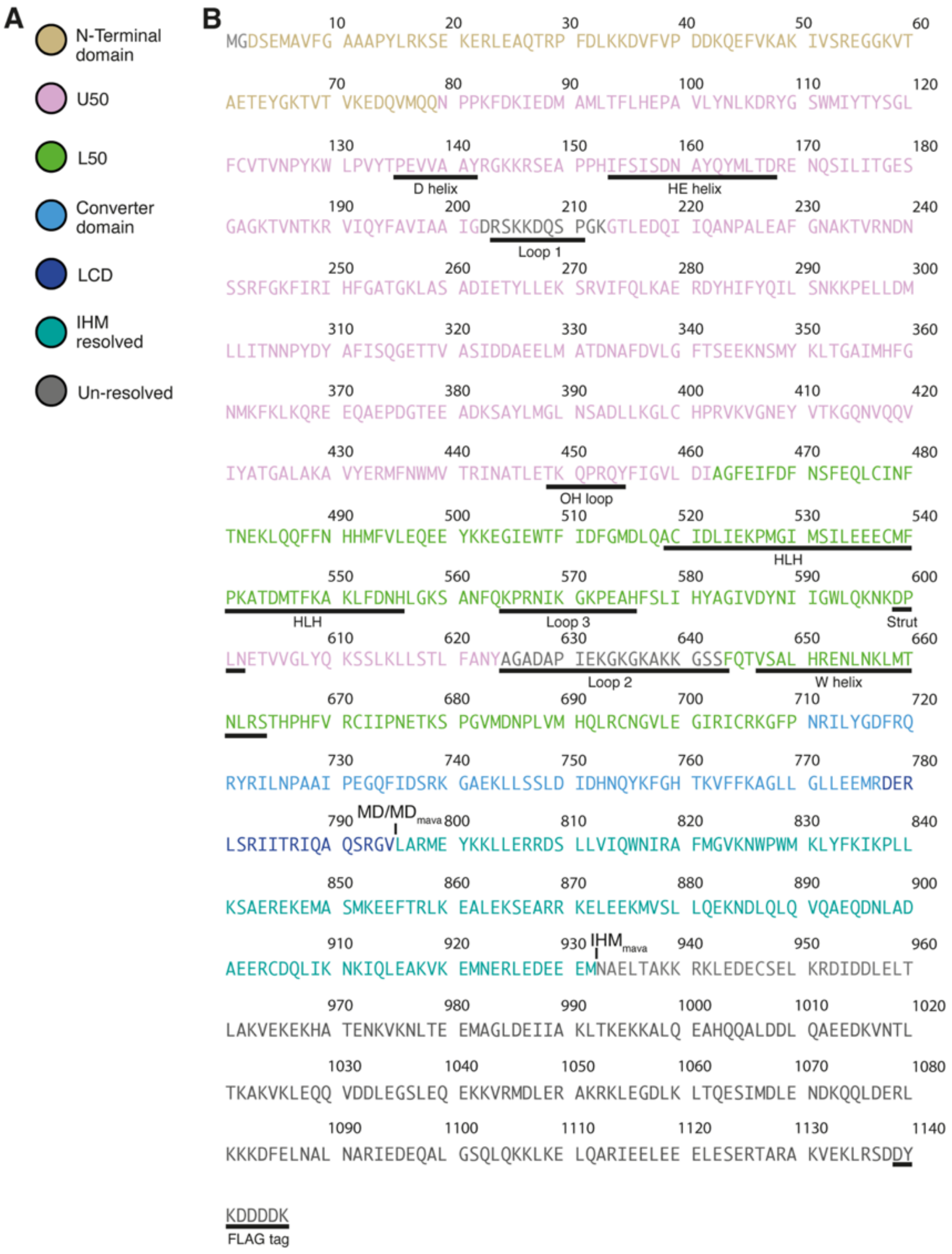

Fig. S3. cHMM heavy chain sequence and subdomains (a) Key for cHMM sequence (b) cHMM heavy chain sequence highlighting resolved sub- domains and key structural regions with C-terminal flag tag.

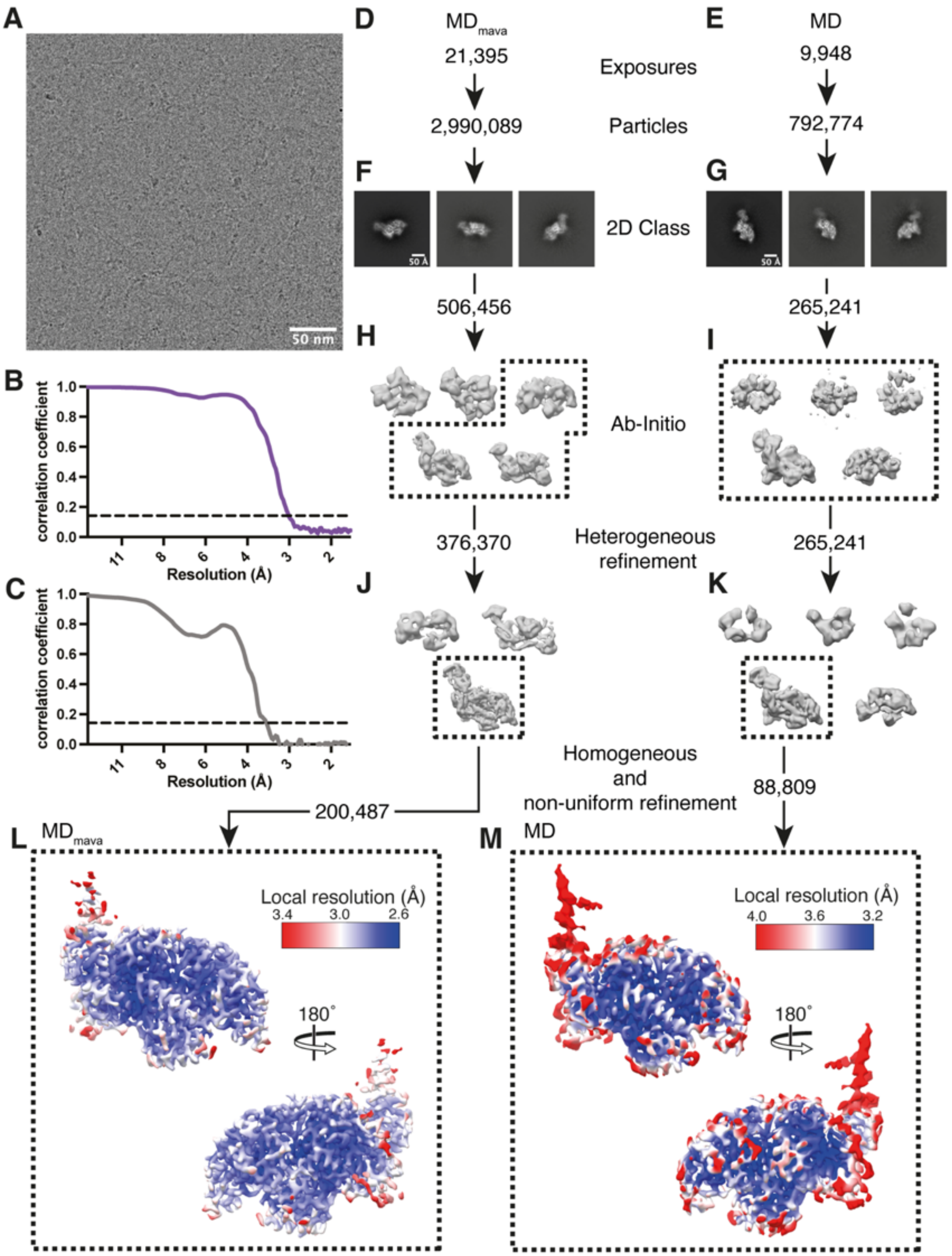

Fig. S4: Open head motor domain processing pipeline. (a) Representative cryoEM micrograph from MD_{mava} dataset. (b-c) FSC curves, MD_{mava} 2.9Å and MD 3.4Å respectively, 0.143 threshold represented by dashed line. (d-m) Flow diagram for MD_{mava} and MD data processing. (d,e) Number for micrographs collected and particles extracted for MD_{mava} and MD respectively (f,g) Representative 2D classes. (h,i) Ab-initio classes, selected classes carried forward for refinement are boxed. (j,k) heterogeneous refinement classes. (l,m) MD_{mava} and MD final reconstruction respectively, coloured by local resolution estimation.

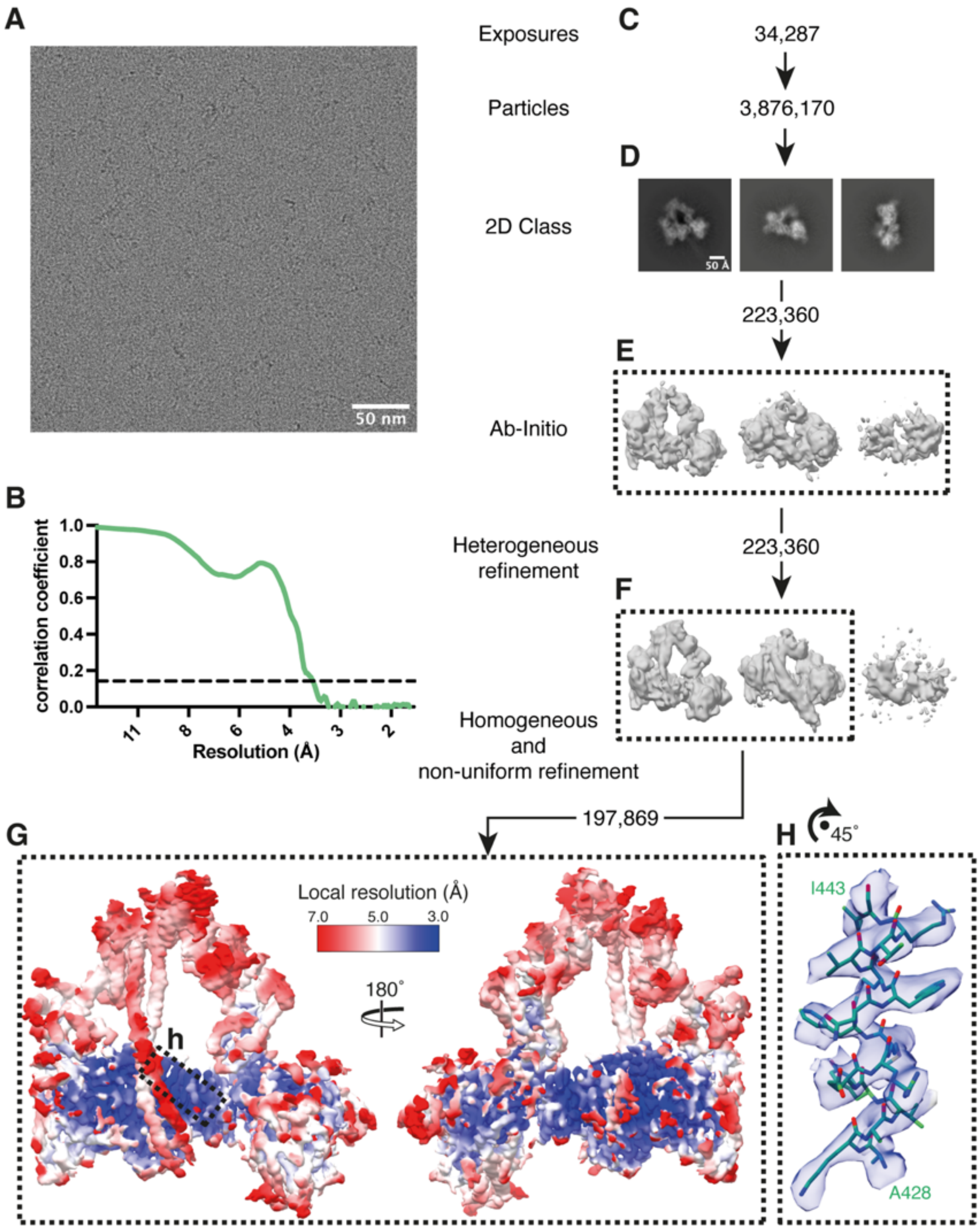

Fig. Fig. S5. IHM$_{mava}$ processing pipeline. (a) Representative cryoEM micrograph from IHM$_{mava}$ dataset. (b) IHM$_{mava}$ FSC curve 3.7Å, 0.143 threshold represented by dashed line. (c-g) Flow diagram for IHM$_{mava}$ image processing. (c) Number of micrographs collected, and particles extracted for IHM$_{mava}$. (d) Representative 2D classes. (e) Ab-initio classes, selected classes carried forward for refinement are boxed. (f) Heterogeneous refinement classes. (g) IHM$_{mava}$ final reconstruction, coloured by local resolution estimation. (h) IHM$_{mava}$ in Segmented cryoEM map highlighting residues A248-I443 in the blocked head, displaying clear side chain density.

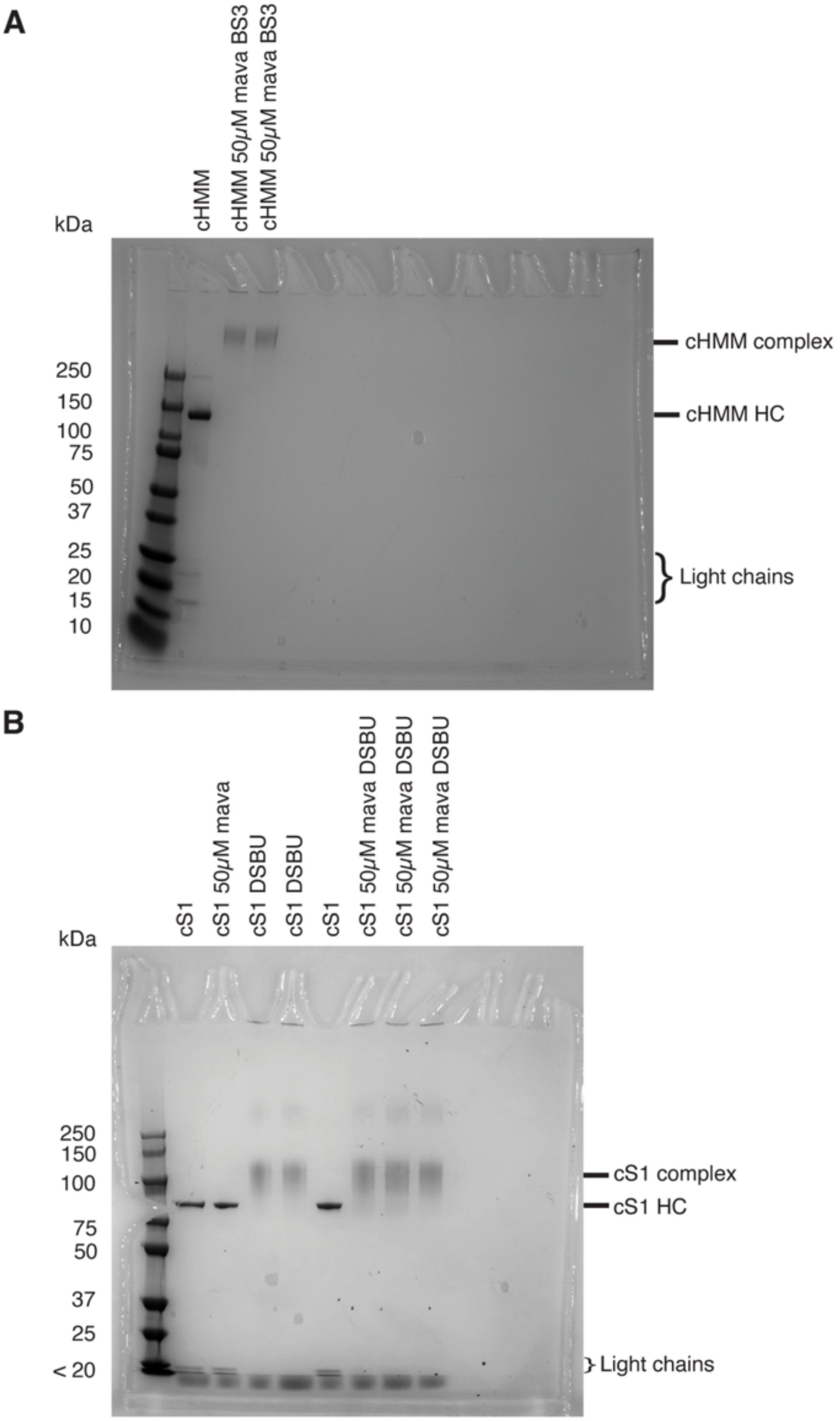

Fig. S6. SDS-PAGE gel of crosslinked cHMM and cS1 complexes. (a) gel shown 4–20% Mini-PROTEAN® TGX™ Precast Protein Gels, 15-well (BioRad) and precision plus dual colour standards (BioRad) ladder with cHMM un-crosslinked and BS3 crosslinked samples highlighting the crosslinked cHMM complex, cHMM heavy chain (HC) and associated light chains. (b) gel shown 3 to 8 % NuPAGE Tris-Acetate Precast Protein Gel with cS1 un-crosslinked and DSBU crosslinked samples highlighting the cS1 complex, HC and LCs.

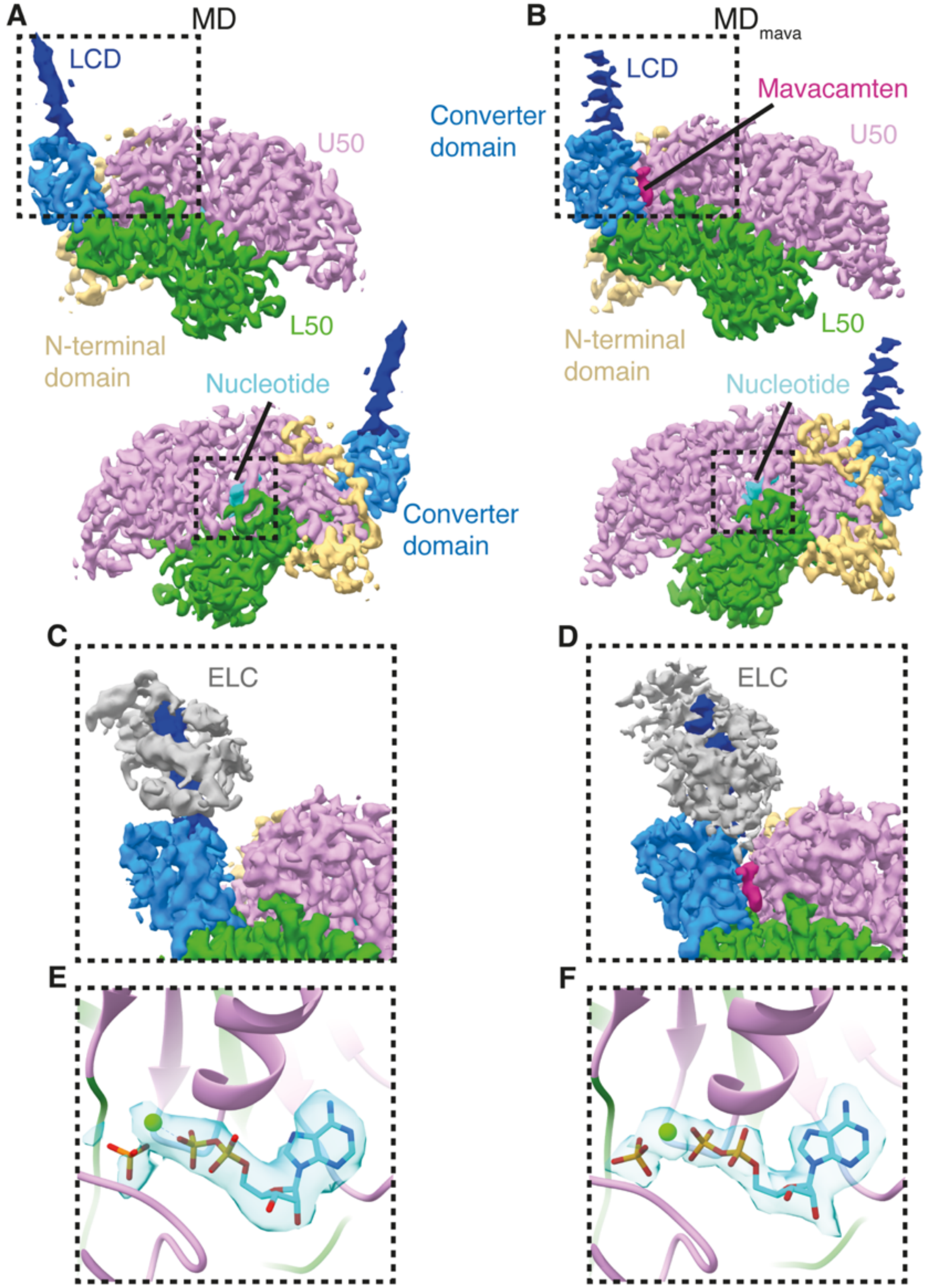

Fig. S7. Primed motor domain cryoEM maps. (a) Segmented cryoEM map MD, split by subdomain (contour 0.5): N-terminal domain beige, L50 green, U50 pink, converter domain light blue and LCD dark blue. (b) Segmented cryoEM map of MD_{mava}, split by subdomain (contour 0.6) coloured as (a) with mavacamten in burgundy. (c-d) Magnified view of segmented cryoEM map lever displaying ELC density grey (c) MD (contour 0.3) (d) MD_{mava} (contour 0.36) (e-f) ADP.P_i fit to segmented density colured by hetroatom (c) MD (contour 0.5) (d) MD_{mava} (contour 0.6).

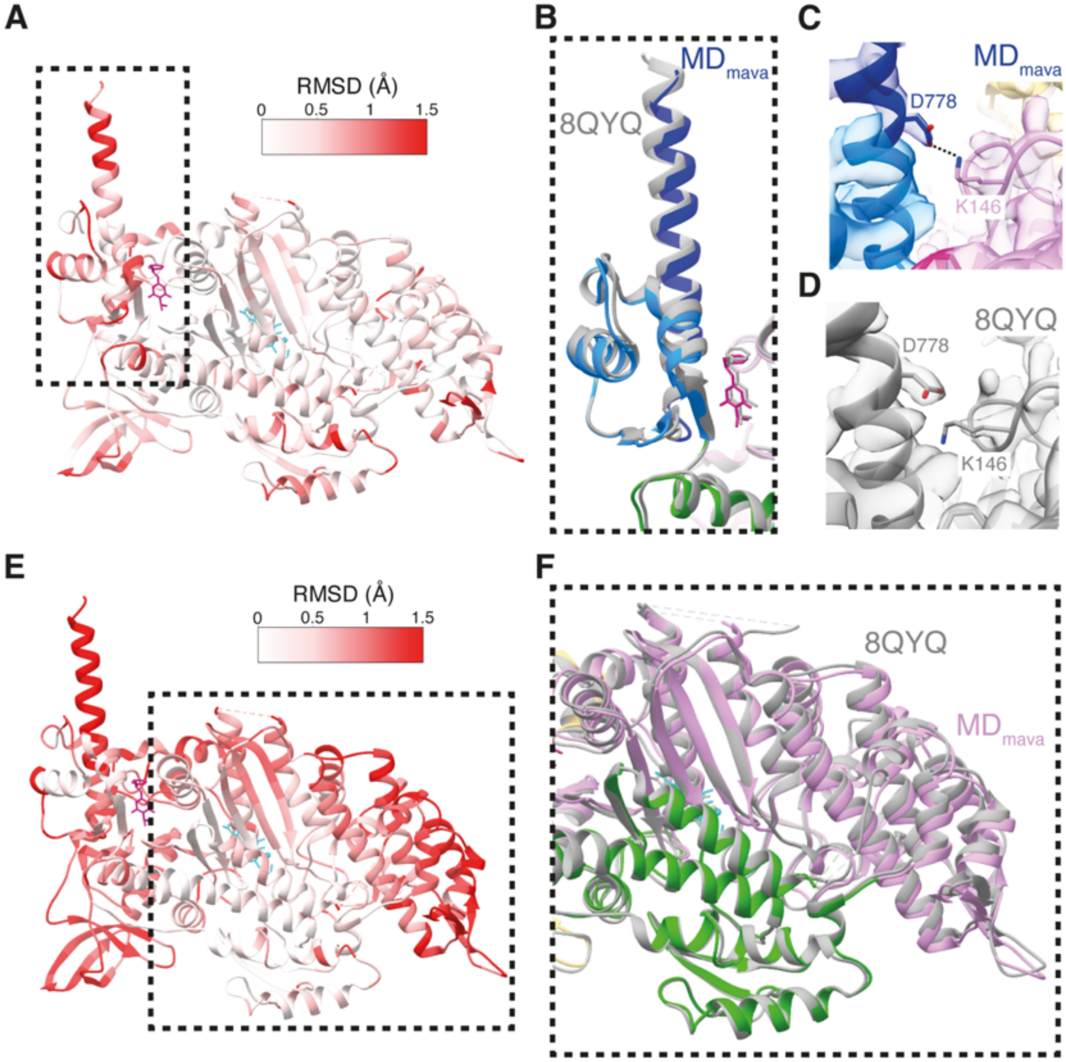

Fig. S8. Comparison of MD$_{mava}$ structure to bovine S1 crystal structure. (a) RMSD comparison between MD$_{mava}$ and crystal structure of bovine S1 in complex with mavacamten (burgundy) (PDB ID: 8QYQ) coloured on MD$_{mava}$ pdb (global structure alignment). (b) Overlay of lever position between MD$_{mava}$, coloured by subdomain (L50 green, U50 pink, converter domain light blue, LCD dark blue and Mavacamten in burgundy) and 8QYQ grey (global structure alignment). (c-d) Magnified view of D778 and K146 interaction in (c) MD$_{mava}$, coloured as in (b) and (d) 8QYQ grey. (e) RMSD comparison as in (a) but aligned using L50. (f) magnified view of (e) displayed as overlay of MD$_{mava}$ coloured as in (b) and 8QYQ grey showing differing U50 conformation.

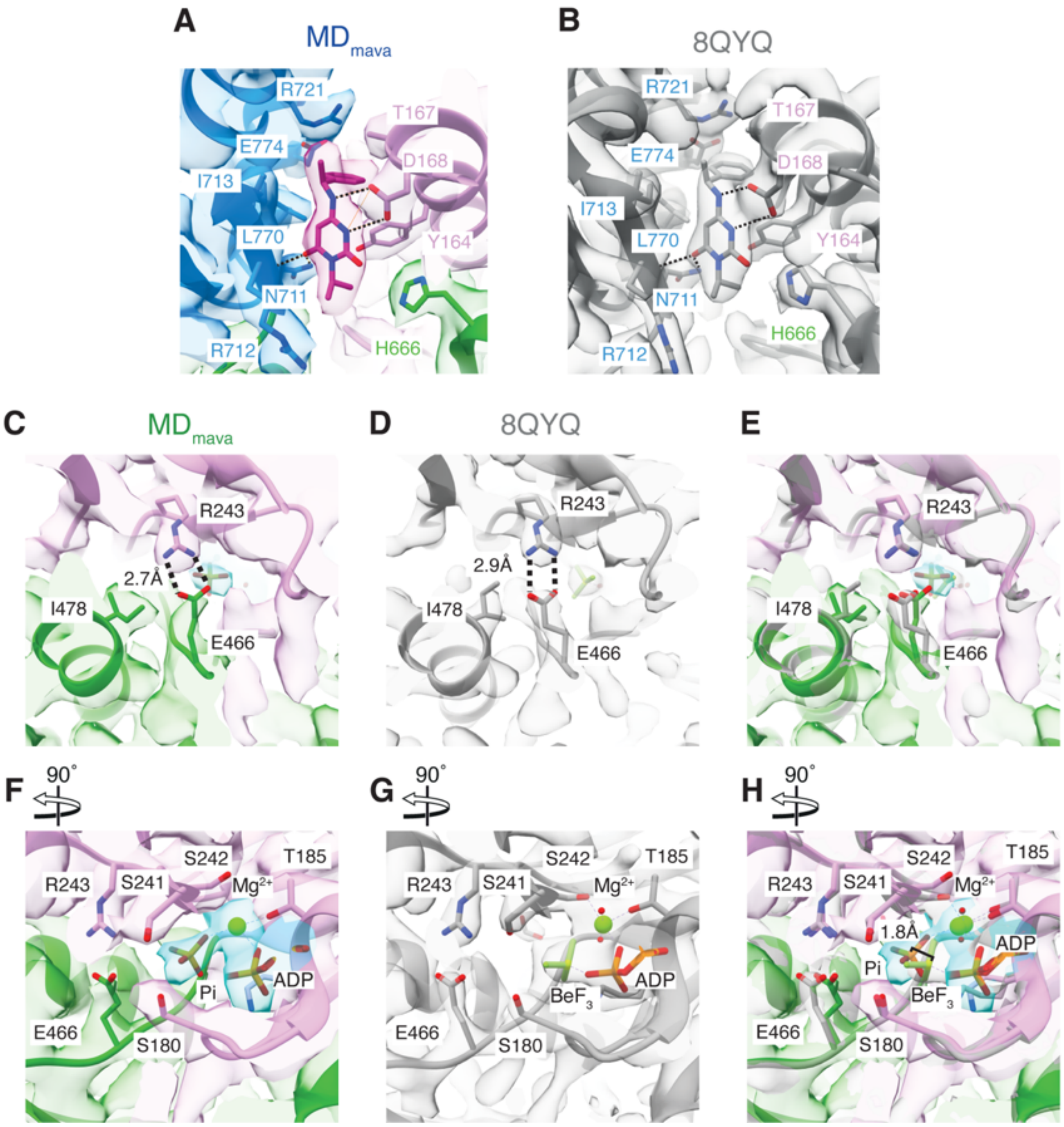

Fig. S9. Comparison of MD_{mava} structure to bovine S1 crystal structure active site. (a-b) Magnified view of mavacamten binding site highlighting interacting residues showing improved compound density in (a) MD_{mava} in segmented cryoEM map (contour 0.6) over (b) 8QYQ in corresponding X-ray crystallography density map (contour 0.15). (c-e) Magnified view of back door residues R243, E466 as well as I478 highlighting differing conformation between (c) MD_{mava} segmented cryoEM map (contour 0.6) and model coloured, (d) 8QYQ crystallographic map (contour 0.3) and model grey and (e) overlay. (f-h) 90° rotation of (c-e) showing P_i/ BeF_3 and surrounding residue positions in (f) MD_{mava}, (g) 8QYQ and (h) overlay highlighting the 1.8 Å difference in Pi and BeF_3 coordination in active site.

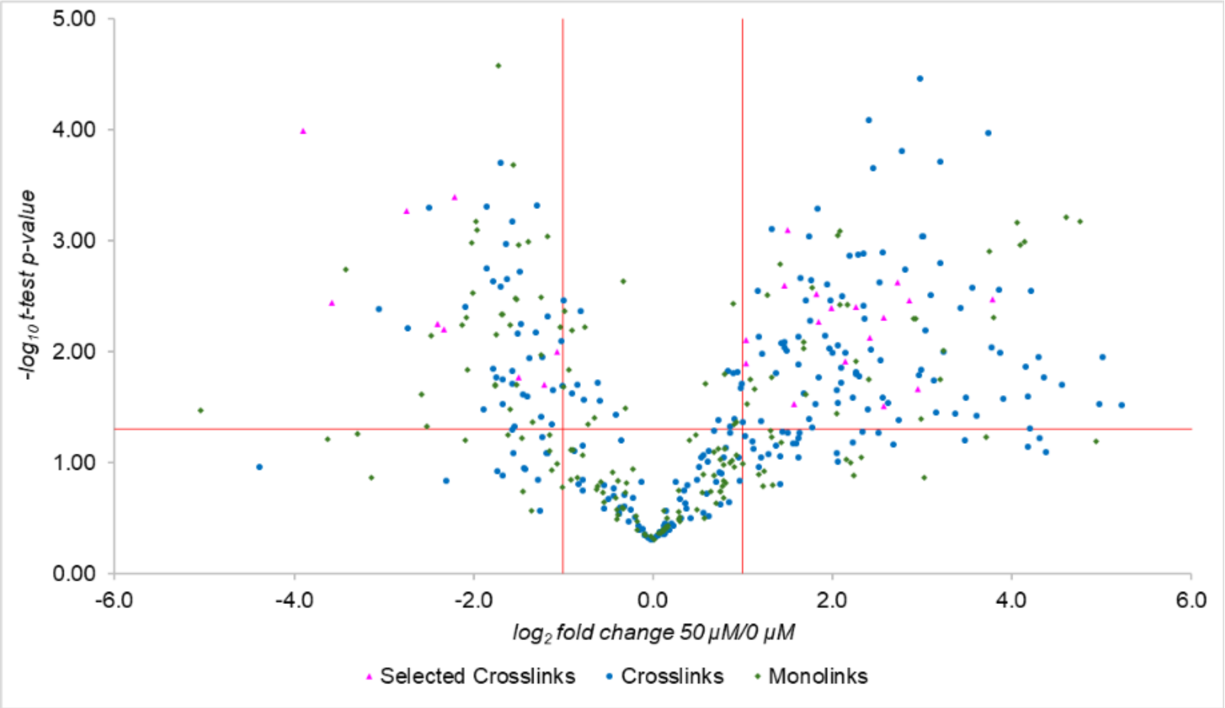

Fig. S10. Volcano plot of -log$_{10}$ t-test p-values versus log2 fold change in crosslinks and monolinks. Crosslinks (▲) described in Supplementary Table 5, and all other crosslinks (•) and monolinks (♦) identified.

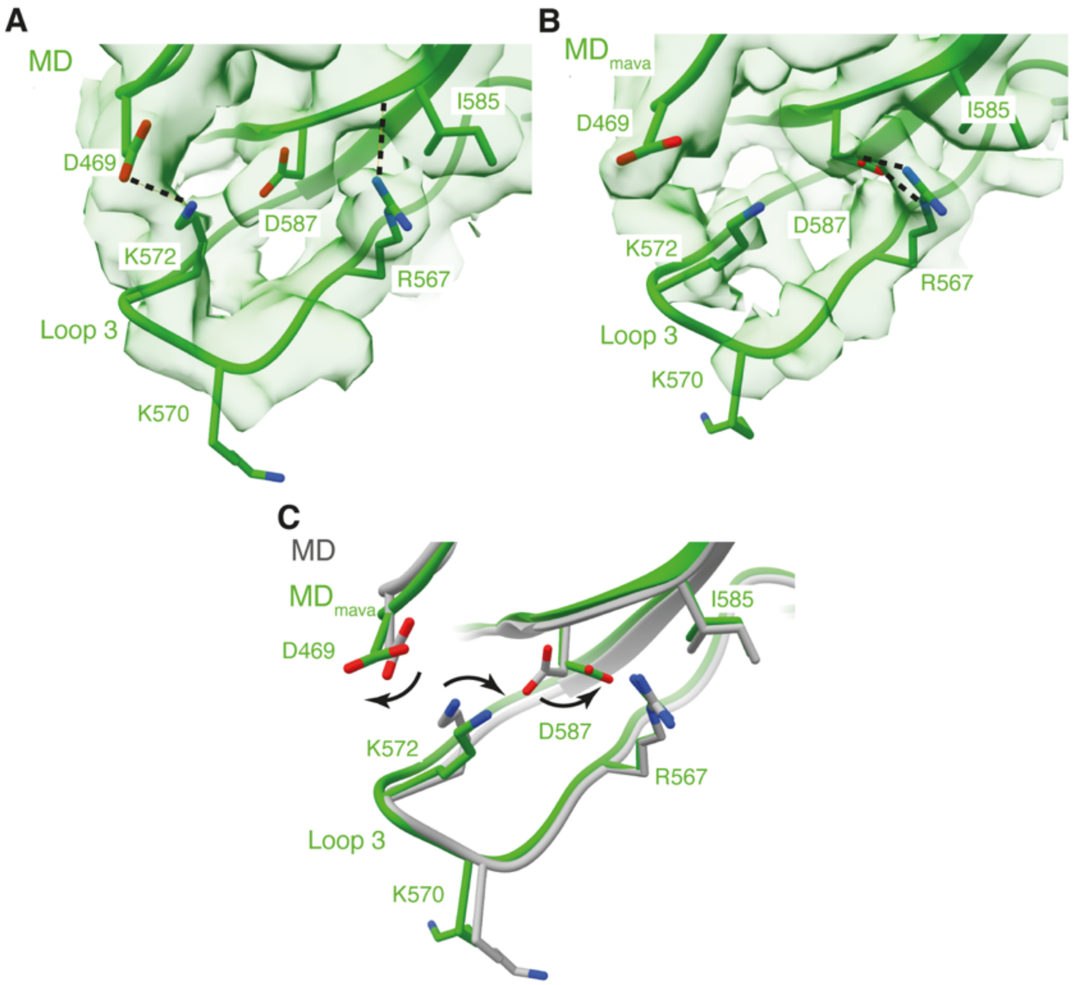

Fig. S11. Allosteric effect of mavacamten on loop 3 hydrogen bonding (a-c) Magnified view of loop 3 hydrogen bonding network. (a) MD model green in segmented cryoEM map (contour 0.25), highlighting hydrogen bonding between D469-K572 and R567-I585 alongside D587 position. (b) MD_{mava} model green in segmented cryoEM map (contour 0.42), highlighting hydrogen bonding between R567-D587 and D469, K572 position. (c) Overlay of MD gray and MD_{mava} green models highlighting change in D469, K572 and D587 resulting in loss of D469-K572 interaction explaining subsequent increase in crosslinking reactivity for K572 in the presence of mavacamten.

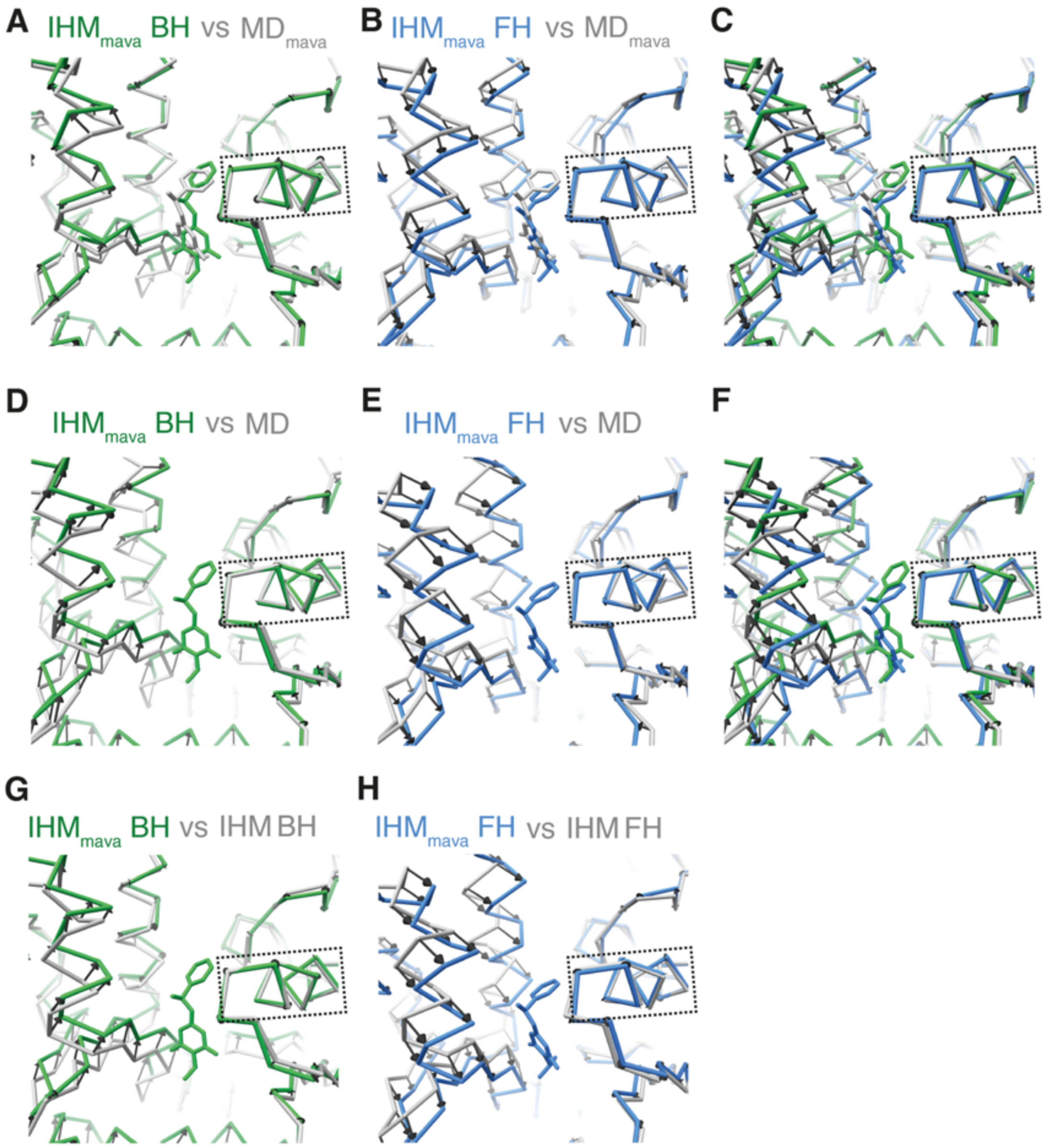

Fig. S12. Change within the mavacamten binding site between the blocked and free head of the IHM. (a-c) Comparison of the MD_{mava} and IHM_{mava} mavacamten binding sites shown as backbone trace with vector arrows between alpha carbons. Models were aligned on the HE helix (residues 154-168) highlighted by the dashed box. (a) MD_{mava} grey and IHM_{mava} BH green. (b) MD_{mava} grey and IHM_{mava} FH blue. (c) Overlay of panels (a-b) to highlight that the conformational change is in opposite directions for the two heads of the IHM. (d-f) Comparison of the MD and IHM_{mava} mavacamten binding sites dispayed as in (a). (d) MD grey and IHM_{mava} BH green. (e) MD grey and IHM_{mava} FH blue. (f) Overlay of (d-e). (g-h) Comparison of the IHM and IHM_{mava} mavacamten binding sites displayed as in (a). (g) IHM BH grey and IHM_{mava} BH green. (h) IHM FH grey and IHM_{mava} FH blue.

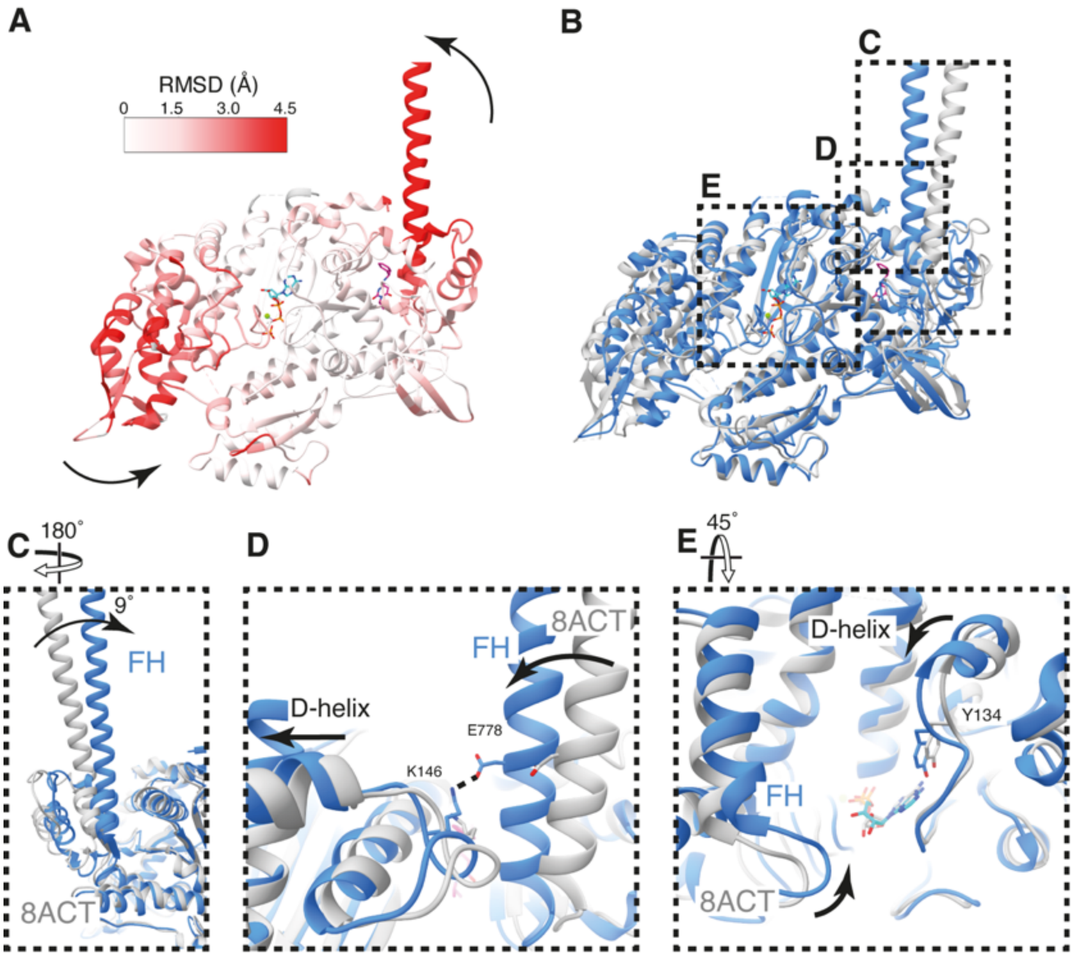

Fig. S13. FH IHM$_{mava}$ comparison to the IHM. (a) RMSD comparison between IHM$_{mava}$ FH and folded-back state FH (PDB ID: 8ACT) aligned on the L50, coloured on IHM$_{mava}$ FH model, highlighting domain movements. (b) Overlay of IHM$_{mava}$ FH blue and folded-back state FH grey. (c) Side view of the IHM$_{mava}$ FH lever overlaid on the folded-back state FH highlighting the 9° shift of the lever, coloured as in (b). (d) Structural comparison of lever and D-helix conformation showing E778-K146 coupling hydrogen bond in IHM$_{mava}$ FH but not in the folded-back state FH, coloured as in (b). (e) Structural comparison of active site highlighting loop closure around active site in IHM$_{mava}$, coloured as in (b).

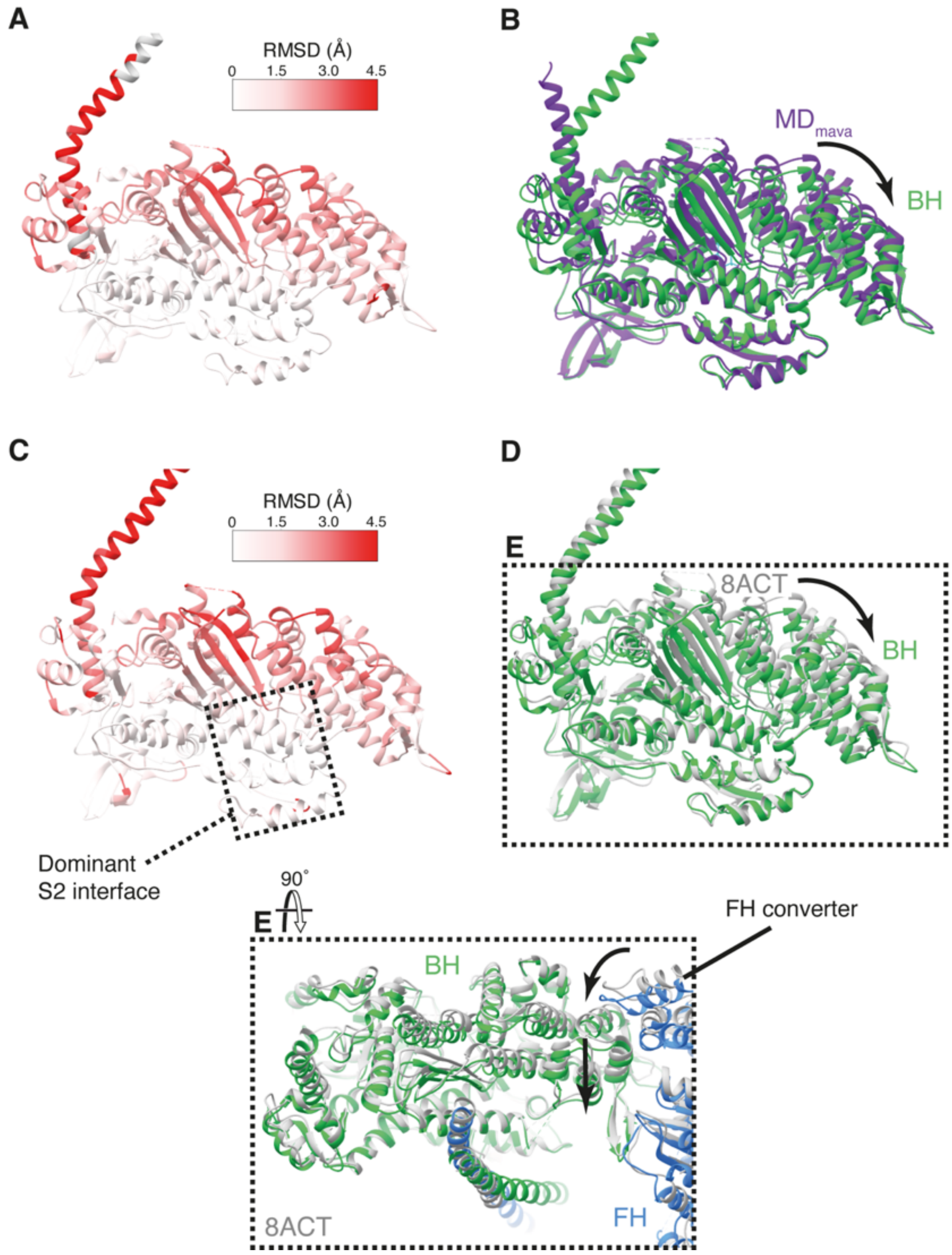

Fig. S14. BH IHM$_{mava}$ comparison to MD$_{mava}$ and the IHM. (a) RMSD comparison between IHM$_{mava}$ BH and MD$_{mava}$ aligned on the L50, coloured on IHM$_{mava}$ BH model (b) Overlay of IHM$_{mava}$ BH green and MD$_{mava}$ purple, highlighting U50 movement. (c) RMSD comparison between IHM$_{mava}$ BH and folded-back state BH (PDB ID: 8ACT) aligned on the L50, coloured on IHM$_{mava}$ FH model, highlighting the region the IHM$_{mava}$ S2 has the most interactions with the BH in our model. (d) Overlay of IHM$_{mava}$ BH (green) and folded-back state BH (grey), highlighting U50 movement. (e) Overlay of IHM$_{mava}$ (green/blue) and folded-back state (grey) aligned on the BH, highlighting how the change in FH lever angle changes BH U50 conformation.

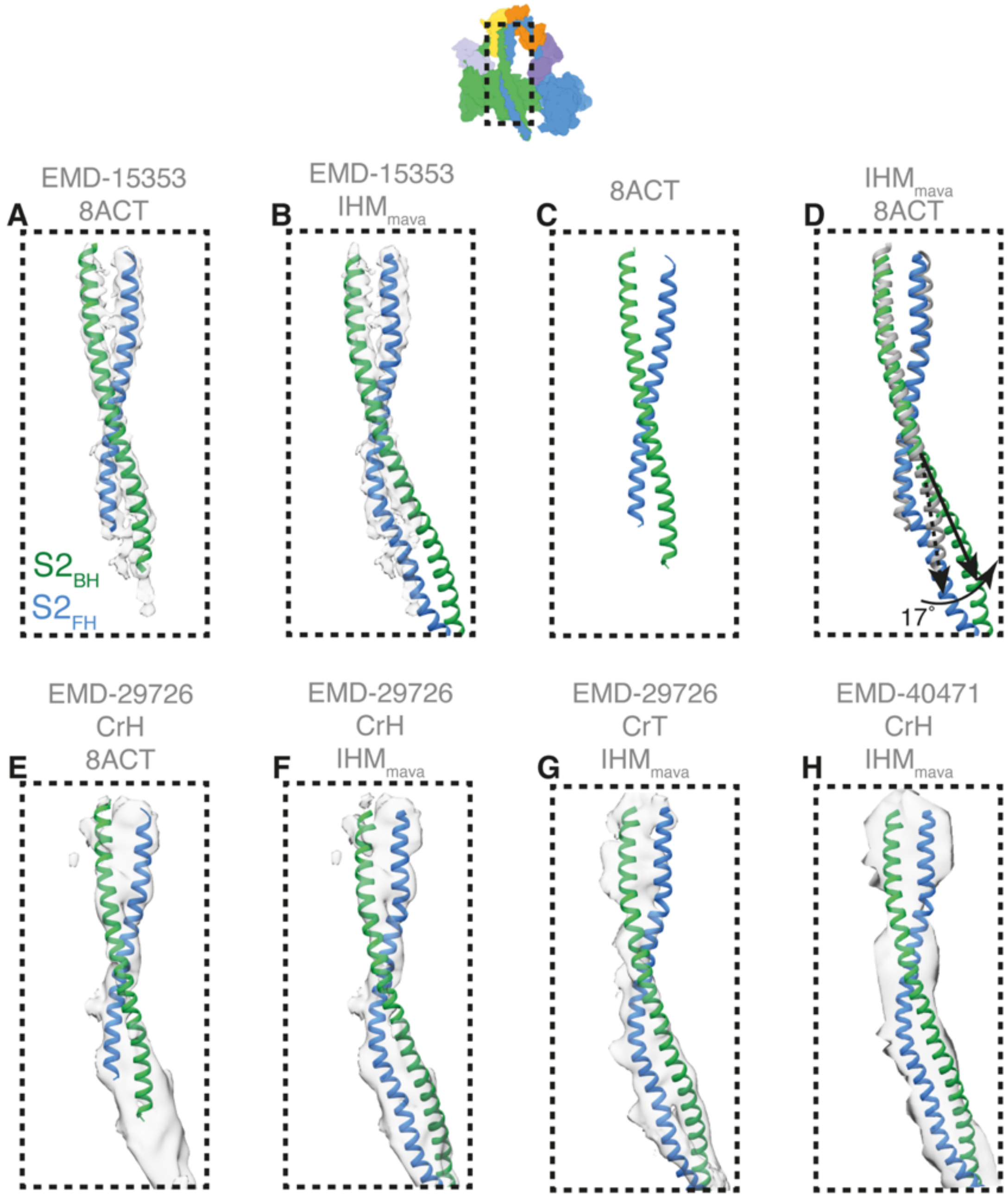

Fig. S15. IHM$_{mava}$ S2 comparison. (a-d) Comparison of S2 conformation between IHM$_{mava}$ and IHM. (a) IHM S2 PDB ID: 8ACT (green/blue) rigidly fitted into corresponding segmented map EMD-15353 (contour 0.2). (b) IHM$_{mava}$ (green/blue) rigidly fitted into segmented map EMD-15353 (contour 0.2). (c) IHM S2 model (green/blue). (d) IHM$_{mava}$ S2 model (green/blue) compared to IHM S2 model (grey) highlighting BH heavy chain angle change of 17° (measured from BH 878-905). (e) Comparison of S2 conformation between IHM and mavacamten stabilised thick filament EMD-29726 horizontal crown (CrH)(contour 0.15) (f-h) Comparison of S2 conformation between IHM$_{mava}$ and mavacamten stabilised thick filament EMD-29726 (f) horizontal crown (CrH)(contour 0.15) and (g) tilted crown (CrT)(contour 0.15).(h) Comparison of S2 conformation between IHM$_{mava}$ mavacamten free thick filament CrH EMD-40471(contour 5.0).

Abbreviation	Definition
ADP.P_i	Adenosine diphosphate and inorganic phosphate
ATP	Adenosine triphosphate
βCM	Beta-cardiac myosin
BH	Blocked head
CON	Converter
cryoEM	Cryogenic electron microscopy
DSBU	Disuccinimidyl dibutyric urea
ELC	Essential light chain
FH	Free head
FDA	Food and Drug Administration
HCM	Hypertrophic cardiomyopathy
HMM (cHMM)	Heavy meromyosin (Cardiac heavy meromyosin)
IHM	Interacting-heads motif
IHM_{mava}	Interacting-heads motif with mavacamten bound
L50	Lower 50-kDa
LCD	Light chain binding domain
LMM	Light meromyosin
MD	Myosin motor domain (ADP.Pi state)
MD_{mava}	Myosin motor domain (ADP.Pi state) with mavacamten bound
NTD	N-terminal domain
P_i	Inorganic phosphate
qXL-MS	Quantitative crosslinking mass spectrometry
RLC	Regulatory light chain
S1 (cS1)	Segment 1 (Cardiac segment 1)
S2	Segment 2
U50	Upper 50-kDa

Table S1. List of abbreviations.

cHMM		cS1	
IC_{50} =	0.14 ± 0.01 μM	IC_{50} =	0.62 ± 0.07 μM
V_o =	1.26 ± 0.04 μm/s	V_o =	2.05 ± 0.05 μm/s
V_i =	1.12 ± 0.04 μm/s	Vi =	1.77 ± 0.07 μm/s
V_{min} =	0.14 μm/s	V_{min} =	0.28 μm/s

Table S2. Inhibition of cardiac myosin motor activity by mavacamten. Where IC_{50} represents the concentration of drug at which actin motility is reduced by 50 %, V_o represents the actin filament speed in the absence of drug, V_i represents the maximum actin filament speed in the presence of drug and V_{min} is the asymptote for inhibition (V_o - V_i).

	MD	MD_{mava}	IHM_{mava}
**Data collection and processing**			
Magnification	96,000 x	96,000 x	96,000 x
Voltage (kV)	300	300	300
Nominal defocus range (µm)	-1.5 to -3	-1.5 to -3	-1.5 to -3
Pixel size (Å)	0.822	0.822	0.822
Total fluence ($e^-/Å^2$)	42.92	43.68<br>43.02	43.49<br>42.92<br>42.96
Exposure time (s)	3.63	3.63<br>3.84	3.95<br>3.63<br>3.78
Number of micrographs	9,948	9,936<br>11,404	9,074<br>13,097<br>12,116
Initial number of particles	3,436,065	6,830,129	3,876,170
	**MD**	MD_{mava}	IHM_{mava}
Final number of particles	88,809	200,487	197,869
Resolution (FSC = 0.143)	3.4 Å	2.9 Å	3.7 Å

Table S3. Data collection and processing statistics for MD, MD_{mava} and IHM_{mava} EM structures. Where multiple collections were combined individual values for each collection are listed.

	MD	MD_{mava}	IHM_{mava}
**Model Refinement**			
Initial Model used	MD_{mava}	6Z47	MD_{mava} + 6Z47
Map-model correlation (FSC = 0.143)	3.4Å	2.9Å	3.7Å
Map-sharpening B-factor (Å^2)	-152.9	-132.3	-151.9
**Model composition**			
Non-hydrogen atoms	6180	6201	19297
Protein residues	764	764	2380
Ligands	1	2	4
**R.M.S.Z deviations**			
Bond lengths (Å)	0.34	0.36	0.33
Bond angles (°)	0.6	0.63	0.65
**Validation**			
MolProbity score	1.13	1.14	1.98
Clashscore	1.38	1.38	14
Poor rotamers (%)	0	0	0
**Ramachandran plot**			
Favoured (%)	96	96	96
Allowed (%)	4	4	4
Disallowed (%)	0	0	0

Table S4. **Model building and refinement statistics for MD, MD_{mava} and IHM_{mava} EM data.**

Protein1	Domain1	Residue1	Protein2	Domain2	Residue 2	Cα-Cα distance MDapo (BHapo)	Cα-Cα distance MDmava (BHmava)	Log2 fold change	T-test	Indication with mava	Figure Panel
MYH7	LCD	K803	RLC	EF-hand 2	K111	21.5	21.5	3.78	0.0034	increased stability	a/c/d
MYH7	U50	K207	RLC	EF-hand 2	K115	*52.1(28.4)*	*45.2 (20.6)*	2.96	0.0218	*increased proximity*	a/c/d
MYH7	L50	K707	MYH7	Converter	K757	13.1	13.1	2.86	0.0034	increased stability	a/e
MYH7	U50	K206	ELC	EF-hand 2	K142	*14.7*	*12.3*	2.73	0.0024	*increased proximity*	a/c/d
MYH7	L50	K707	MYH7	Converter	Y715	22.5	22.3	2.57	0.0308	increased stability	a/e
MYH7	LCD	K835	RLC	EF-hand 3	K165	9.5	9.5	2.57	0.0049	increased stability	a
MYH7	NTD	K34	MYH7	U50	K83	15.9	15.7	2.52	0.0024	increased stability	a/e
MYH7	U50	K206	RLC	EF-hand 2	K115	*50.2 (30.5)*	*44.2 (22.6)*	2.41	0.0075	*increased proximity*	a/c/d
MYH7	U50	K207	ELC	EF-hand 2	K142	*18.3*	*15.5*	2.26	0.0151	*increased proximity*	a/c/d
MYH7	U50	K405	MYH7	L50	K598	18.1	17.6	2.26	0.0039	increased stability	a/f
MYH7	LCD	K825	RLC	EF-hand 3	K165	19.2	19.2	2.15	0.0122	increased stability	a
MYH7	LCD	K825	RLC	EF-hand 2	K91	13.8	13.8	1.85	0.0054	increased stability	a
MYH7	U50	K278	MYH7	L50	K679	18.4	19.0	1.83	0.0030	increased stability	a/f
MYH7	U50	K257	ELC	EF-hand 2	K142	*16.7*	*13.4*	1.57	0.0298	*increased proximity*	a/c/d
MYH7	NTD	K34	MYH7	L50	K707	25.6	24.5	1.51	0.0008	increased stability	a/e
MYH7	LCD	K803	RLC	EF-hand 2	K115	21.4	21.4	1.47	0.0025	increased stability	a/c/d
MYH7	LCD	K835	RLC	EF-hand 1	K62	18.4	18.4	1.04	0.0126	increased stability	a
MYH7	U50	K413	MYH7	L50	K598	19.4	19.3	1.04	0.0078	increased stability	a/f
MYH7	NTD	K50	MYH7	L50	K707	29.9	28.8	-1.07	0.0099	reduced reactivity	b/e
MYH7	NTD	K21	ELC	EF-hand 1	Y73	40.4	40.3	-1.21	0.0199	reduced dynamics	b/e
MYH7	L50	K707	ELC	EF-hand 1	Y73	57.7	57.5	-1.50	0.0170	reduced dynamics	b/e
MYH7	U50	K450	MYH7	L50	K707	45.6	45.6	-2.04	0.0000	reduced dynamics	b/f
MYH7	NTD	K21	ELC	EF-hand 1	K98	28.3	27.9	-2.21	0.0004	reduced reactivity	b/e
ELC	EF-hand 1	K66	RLC	EF-hand 2	K104	30.9	30.8	-2.33	0.0063	reduced dynamics	b
MYH7	NTD	K35	ELC	EF-hand 1	K98	47.4	48.9	-2.40	0.0057	reduced dynamics	b/e
MYH7	U50	K450	MYH7	L50	K657	24.5	24.8	-3.57	0.0036	reduced reactivity	b/f
MYH7	NTD	K50	ELC	EF-hand 1	K98	49.9	51.0	-3.90	0.0001	reduced dynamics	b/e

Table S5. Interdomain crosslinks annotated on Fig. 3. Cα-Cα distances that change >2 Å between models are shown in *italics*. Positive to negative log2fold change in crosslink intensity on addition of mavacamten is indicated on a colour scale from red to blue.

Movie S1. Mavacamten reduces the number of moving filaments in cHMM and not cS1. Movie demonstrates actin gliding movment displayed as summed plots of actin filament movement over 100 seconds of motility in Fig. 1b,c. Actin gliding is shown for cHMM and cS1 +/- 5 μM mavacamten, each panel correspond to 100 sec of movement captured at 5 frames/sec. Playback is at 20 fps (4x speed) to illustrate the slow movement at 5 μM mavacamten. Without drug the actin filaments glide smoothly over both cHMM and cS1 surfaces. However, at saturating [mavacamten] (5 μM) the fraction of moving filaments for cHMM decreases and movement is often interrupted by long pauses. Comparatively, filaments continue to move smoothly over the cS1 surface with only a reduction in speed.

Movie S2. Structural changes induced by mavacamten binding open motors. Overview of key structural changes in the motor domain induced by mavacamten binding. Model state and morph direction is shown in the top left of the movie. (0:00) Overview of MD segmented cryoEM map (contour 0.5) split by subdomain: N-terminal domain beige, L50 green, U50 pink, converter domain light blue and LCD dark blue. (0:14) 360° rotation of MD PDB in segmented map. (0:30) Fade to un split sharpened MD cryoEM map (contour 0.56) and representation of mavacamten binding. (0:35) Morph of map and pdb from MD to MD_{mava}. (0:44) Conformational changes induced by mavacamten binding, MD pdb grey and MD_{mava} pdb coloured. (0:58) 360° rotation of MD_{mava} pdb in segmented cryoEM map (contour 0.6) split by subdomain coloured as in (0:00) as well as Mavacamten in burgundy. (1:17) Magnified view of mavacamten binding site. (1:35) magnified view of LCD D-helix interaction. (1:39) Fade to un split sharpened MD_{mava} cryoEM map (contour 0.56) followed by morph of map and model from MD_{mava} to MD and back. (1:51) Highlighting conformational change at the D-helix between MD grey and MD_{mava} coloured. (2:17) Magnified view of MD_{mava} back door pdb in segmented cryoEM map shown as in (0:58). (2:20) Fade to un split sharpened MD_{mava} cryoEM map (contour 0.56) followed by morph of map and model from MD_{mava} to MD and back. (2:34) Highlighting conformational change at the back door between MD grey and MD_{mava} coloured.

Movie S3. IHM_{mava} interaction interfaces. (0:00) Overview and 360° rotation of IHM_{mava} segmented cryoEM map coloured by chain (contour: 0.08): blocked head green, free head blue, blocked head ELC light purple, free head ELC purple, blocked head RLC yellow, free head RLC orange, mavacamten burgundy and the nucleotide in light blue. (0:31) 360° rotation of IHM_{mava} PDB in segmented cryoEM map coloured as in (0:00). (0:50) Magnified view of BH mavacamten binding site. (1:07) Magnified view of FH mavacamten binding site. (1:30) Magnified view of motor-motor interface (change in map contour to 0.01). (1:46) Magnified view of HCM $loop_{BH}$ $transducer_{FH}$ interface. (2:03) Magnified view of BH ELC_{FH} interface. (2:22) Overview of S2 BH interface highlighting the three main contact regions on the BH: OH-helix, W-helix and HLH. (2:36) Magnified view of S2 OH-$helix_{BH}$ interface. (2:49) Magnified view of S2 W-$helix_{BH}$ interface. (3:01) Magnified view of S2 HLH_{BH} interface.

Data S1. (separate file)

Excel spreadsheet detailing crosslinks and monolinks that changed significantly in the presence or absence of mavacamten with significance determined by pair comparison via a single tailed, homoscedastic t-test using relative signal intensity, protein fold changes >2 and $p < 0.05$.

Data S2. (separate file)

Excel spreadsheet detailing quantitative assessment of crosslinking mass spectrometry data.
